# A Multicomponent, Preschool to Third Grade Preventive Intervention and Educational Attainment at 35 Years of Age

**DOI:** 10.1001/jamapediatrics.2017.4673

**Published:** 2018-01-29

**Authors:** Arthur J. Reynolds, Suh-Ruu Ou, Judy A. Temple

**Affiliations:** 1 Institute of Child Development, University of Minnesota, Minneapolis; 2 Humphrey School of Public Affairs, University of Minnesota, Minneapolis

## Abstract

**Importance:**

Educational attainment is the leading social determinant of health, but few studies of prevention programs have examined whether the programs are associated with educational attainment outcomes after the mid-20s, especially for large-scale programs that provide a longer duration of services.

**Objective:**

To examine the association between a preschool to third grade intervention and educational attainment at midlife and differences by program duration, sex, and parental educational level.

**Design, Setting, and Participants:**

This matched-group, alternative intervention study assessed 1539 low-income minority children born in 1979 or 1980 who grew up in high-poverty neighborhoods in Chicago, Illinois. The comparison group included 550 children primarily from randomly selected schools participating in the usual early intervention. A total of 989 children who entered preschool in 1983 or 1984 and completed kindergarten in 1986 were included in the Chicago Longitudinal Study and were followed up for 27 to 30 years after the end of a multicomponent intervention. A total of 1398 participants (90.8%) in the original sample had educational attainment records at 35 years of age. The study was performed from January 1, 2002, through May 31, 2015.

**Interventions:**

The Child-Parent Center Program provides school-based educational enrichment and comprehensive family services from preschool to third grade (ages 3-9 years).

**Main Outcomes and Measures:**

Educational outcomes from administrative records and self-report included school dropout, 4-year high school graduation, years of education, postsecondary credential, and earned degrees from associate’s to master’s or higher.

**Results:**

A total of 1539 participants (mean [SD] age, 35.1 [0.32] years; 1423 [92.9%] black and 108 [7.1%] Hispanic) were included in the study. After weighting on 2 propensity scores, preschool participants had higher rates of postsecondary degree completion, including associate’s degree or higher (15.7% vs 10.7%; difference, 5.0%; 95% CI, 1.0%-9.0%), master’s degree (4.2% vs 1.5%; difference, 2.7%; 95% CI, 1.3%-4.1%), and years of education (12.81 vs 12.32; difference, 0.49; 95% CI, 0.20-0.77). Duration of participation showed a consistent linear association with outcomes. Compared with fewer years, preschool to second or third grade participation led to higher rates of associate’s degree or higher (18.5% vs 12.5%; difference, 6.0%; 95% CI, 1.0%-11.0%), bachelor’s degree (14.3% vs 8.2%; difference, 6.1%; 95% CI, 1.3%-10.9%), and master’s degree or higher (5.9% vs 2.3%; difference, 3.6%; 95% CI, 1.4%-5.9%). The pattern of benefits was robust and favored male participants for high school graduation, female participants for college attainment, and those from lower-educated households.

**Conclusions and Relevance:**

This study indicates that an established early and continuing intervention is associated with higher midlife postsecondary attainment. Replication and extension of findings to other locations and populations should further strengthen confidence in the health benefits of large-scale preventive interventions.

## Key Points

**Question:**

Does participation in a large-scale, preschool to third grade intervention for economically disadvantaged children link to midlife educational attainment?

**Findings:**

In a cohort study that followed up 1398 children to 35 years of age, 4 to 6 years of intervention was significantly associated with a 48% higher rate of degree completion (associate’s degree or higher) compared with lesser participation. Preschool participation was independently associated with most attainment outcomes, including years of education, with greater benefits for those whose mothers were high school dropouts.

**Meaning:**

Multiyear, comprehensive preventive interventions beginning in early childhood can promote long-term educational success that contributes to positive health and economic outcomes.

## Teaser

This cohort study examines the association between a preschool to third grade intervention with educational attainment at midlife and differences by program duration, sex, and parental educational level.

## Introduction

Because of its influence on a wide variety of life-course outcomes, educational attainment is arguably the most important long-term outcome of early childhood interventions (ECIs). Low educational attainment (ie, high school credential or less) is a major risk factor for all 7 health metrics of the American Heart Association (eg, hypertension, smoking, and obesity),^1,2,3^ economic disparities, criminal behavior, and mental health problems.^4,5,6,7,8^ For these reasons, educational attainment is the leading social determinant of health in Healthy People 2020.^9^ Early childhood interventions are one of the most promising and consequential of all prevention programs,^10,11,12,13^ but few, if any, studies have examined the entire spectrum of education from high school dropout to postsecondary success, primarily because of the lack of follow-up beyond 25 years of age, when continuing education is prevalent.

Findings are also inconsistent across studies,^14,15,16,17,18,19^ with some showing positive effects on high school graduation and/or college attendance and others showing no such effects, a mixed pattern favoring high school or college attainment but not both, and differences by sex or racial groups. Moreover, to our knowledge, no previous studies have assessed degree completion for large-scale, public programs after the mid-20s.

In the Perry Preschool Study,^17^ for example, program participants had significantly higher rates of high school graduation than controls, but no differences were observed for postsecondary degree attainment. The reverse pattern was found in the Carolina Abecedarian Project.^18^ Because of small sample sizes, no credible interpretations about subgroup associations were possible. A designed replication of the Abecedarian Project, the Infant Health and Development Program, found no differences in high school dropout.^16^ Other long-term follow-up studies, including of Head Start,^15,19^ have reported positive associations with educational attainment up to 25 years of age. Differences by sex vary across studies,^17,18,19,20^ but high-risk groups, including the most economically disadvantaged, have had greater benefits.^13,20,21^

Given the relatively small samples and limited number of programs analyzed in long-term follow-up studies, differences by child and family characteristics and for different dosages of intervention are underinvestigated.^22,23^ This limits the implications of findings for health promotion and economic mobility. Because postsecondary attainment for low-income, minority populations is not usually completed until the early 30s,^24^ midlife follow-up is essential for understanding impacts. Sex and family socioeconomic status moderate life-course outcomes.^4,15,24^

In our study, we assessed for the first time, to our knowledge, links between participation in the Child-Parent Center (CPC) program, a large-scale and multicomponent school-based ECI, and educational attainment at 35 years of age. We addressed whether (1) CPC program participation beginning at 3 or 4 years of age was linked to greater educational attainment, including postsecondary degree completion; (2) duration of participation up to 6 years was associated with greater attainment; and (3) increases were modified by sex and parental educational level. We hypothesized that earlier and continuing participation would link to attainment and that men and those whose parents had less education would show greater benefits.

## Methods

### Study Design

The Chicago Longitudinal Study (CLS) is a multisite, prospective investigation of CPC and early experiences on well-being across the life course.^14,25,26^ The study sample includes 1539 low-income, minority children born in 1979 or 1980 who grew up in high-poverty neighborhoods in Chicago, Illinois. In this matched-group, alternative-intervention design, all 989 children who entered preschool in 1983 or 1984 and completed kindergarten in 1986 from 20 CPCs were included. The same-age comparison group of 550 children was enrolled from 5 randomly selected Chicago public schools with alternative ECIs (full-day kindergarten; 15% with Head Start preschool) or 8 CPC sites without preschool experience. Previous reports^25,26,27^ have described the sampling, baseline characteristics, and design elements (eTable 1 and eTable 2 in the Supplement). All data collection procedures have been approved by the University of Minnesota Institutional Review Board. All participants provided written or oral informed consent, and all data were deidentified for analysis. 

Because of living in high-poverty neighborhoods, children in this cohort were eligible for and participated in government-funded early childhood programs. Like most other studies of established programs, random assignment to the intervention was not possible and would have violated the legal rules that require enrollment of the neediest children on a first-come, first-serve basis.^25,27^ Data were collected from administrative records, schools, and families from birth to 35 years of age.

### Intervention

Because the CPC program has been described in depth elsewhere,^21,25,27^ herein, we provide an overview. The CPC program is a school-based preventive intervention for preschool- and school-aged children in high-poverty neighborhoods that are not being otherwise served. The program is designed to promote children's school competence, especially school readiness, achievement, and family involvement in learning. Each center is colocated in a school or on school grounds and serves 100 to 150 children beginning in preschool who then matriculate to school-age services in the same location. Any child who visits a CPC site receives services. Participation age ranges from 1 to 6 years. In addition to a leadership team in each site, all teachers have bachelor’s degrees and are state certified (eMethods in the Supplement).

Major program elements include the following: (1) high-quality educational enrichment through reduced class sizes and a balance of teacher- and child-directed learning; (2) family support services that include participation in school activities, support groups and workshops, and home visits; and (3) comprehensive services that include nutritional and health supports (ie, subsidized meals, health screening, and speech therapy). Service continuity from preschool to third grade (ages 3-9 years) is a hallmark, thus providing a stable and predictable learning environment. The CPC program is being expanded as a multicomponent school reform model.^28,29^

### Outcomes

The full spectrum of secondary and postsecondary education was assessed. All measures were dichotomous except years of education. Data were obtained from administrative records of various sources and were supplemented by interviews with youths and family members. For higher education, the National Student Clearinghouse was the primary source.^30^ More than 3600 public and private colleges, representing 98% of all students, report enrollment and graduation data. We used data collected from matched records from those reports and the Illinois Shared Enrollment and Graduation Consortium (now part of the National Student Clearinghouse) from January 1, 2002, to May 31, 2015 (mean age, 35.1 years) (eMethods in the Supplement). The amount and sources of information were similar among the intervention groups.

Secondary education was measured by 5 indicators: dropout by 16 years of age, 4-year graduation (earned diploma within 4 years of ninth grade entry), and 3 others, including high school completion (earned diploma or General Educational Development credential). Postsecondary education was measured by years of education (range, 7-22 years), college attendance (2 or 4 years), degree completion (associate’s and higher), and a credential (eg, occupational certificate) (eMethods in the Supplement).

### Statistical Analysis

With use of methods of previous studies,^14,21,25^ outcomes were analyzed by probit and linear regression in Stata statistical software, version 14 (StataCorp).^31^ Findings are reported as means or percentages and group differences after adjusting for the influence of the covariates through inverse probability weighting (IPW).^32,33^ Inverse probability weighting adjusted for each individual’s propensity to enroll in the program (selection model) and to leave the study sample (attrition model) (eMethods in the Supplement). After major assumptions were corroborated, propensity scores for each model were estimated through probit regression, with the resulting values used as inverse weights to estimate associations.^34^ As a double adjustment, the weights were multiplied together.

Inverse probability weighting is a more flexible and comprehensive analytic approach than traditional covariate adjustment or matching and yields estimates with the lowest variances and SEs in large samples while correcting for potential biases.^34,35^ The covariates of the propensity scores were measured primarily between birth and 5 years of age, including family risk indicators, sex, race/ethnicity, and child welfare history. There were 17 covariates for the selection model and 26 for the attrition model (eTables 3 and 4 in the Supplement).

Models were estimated separately for preschool and school-age and for extended intervention groups. Dichotomously coded CPC preschool participation at 3 or 4 years of age and school-aged participation from first to third grade were assessed simultaneously. The CPC extended intervention for 4 to 6 years was assessed against the comparison group with less extensive participation (0-3 years). Duration was also analyzed among 6 groups consistent with a dosage-response relationship.

A dummy code for missing data was included to determine whether estimates based on multiple imputation (expectation maximization) affected outcomes. We adjusted SEs for school-level clustering (mean intraclass *r* value, 0.05), although this did not affect estimates. Group differences and marginal effects were emphasized, with 95% CIs. Robustness analyses included each IPW model separately (selection and attrition) and the traditional covariate approach. Sex and mother’s educational level subgroups were estimated in separate models, with interpretations emphasizing differences shown in the overall model.

## Results

### Cohort Follow-up at 35 Years of Age

A total of 1539 participants (mean [SD] age, 35.1 [0.32] years; 1423 [92.9%] black and 108 [7.1%] Hispanic) were included in the study. In May 2015, a total of 1398 participants (90.8%) from the original sample had data on educational attainment. Retention rates were 91.4% for the intervention group and 89.8% for the comparison group. The high rate of retention was attributable to the use of multiple sources of data, including administrative, self-report, and follow-up tracking. Among those with available information, 963 (72.5%) in the total sample resided in Illinois, with most others (213 [16.0%]) remaining in the Midwest (70 participants had missing contact information). Table 1 gives the rates of participation over time and attrition. The comparison group received a variety of early childhood services. There was no evidence of selective attrition by program status.^14,25^ The attrition sample included those who moved and were not located or deceased (eTable 1 and eMethods in the Supplement).

**Table 1.** Participation of Original Child-Parent Center (CPC) Program and Comparison Groups in the Chicago Longitudinal Study

Study Category	Total Sample	Program Group^a^	Comparison Group^a^
Program participant characteristics at start of study^b^			
No. in original sample	1539	989	550
No. with preschool participation	1073	989	84
No. with CPC preschool	989	989	0
Years in CPC preschool (range)	0.99 (0-2)	1.55 (1-2)	0
No. with kindergarten participation	1539	989	550
No. in full-day kindergarten	1141	591	550
No. with CPC school-aged participation	850	684	166
Years in school-aged program (range)	1.16 (0-3)	1.43 (1-2)	0.68 (1-2)
No. with CPC extended intervention (4-6 y)	553	553	0
Total years in CPC program (range)	2.78 (0-6)	3.95 (1-2)	0.68 (1-2)
No. unavailable for follow-up in postprogram years			
Moved^c^	75	41	34
Deceased	66	44	22
Follow-up study at 35 y of age, No. with data			
Educational attainment	1398	904	494
Completed high school (diploma or equivalent)	1157	774	383
Attended college (2- or 4-y institution)	813	555	258
Earned associates’ degree or higher	239	176	63
Earned bachelors’ degree or higher	167	124	43

^a^ Program participation covers the 6-year period (1983-1989) that defines enrollment in the CPC intervention.

^b^ The CPC preschool comparison group participated in a full-day kindergarten program, and 84 participated in Head Start preschool. A total of 109 parents in the comparison group reported that their child participated in other child care or education in preschool, and 176 individuals in the comparison group were eligible to receive limited services in the CPC program for kindergarten but enrolled in different classrooms; they are not part of the original CPC intervention group. Some individuals in the comparison group participated in the school-aged program because it was open to any child enrolled in elementary school from first to third grade. Fifteen children in the CPC intervention group enrolled in the alternative full-day kindergarten.

^c^ These categories account for attrition from the original study sample of 1539. Individuals were unavailable for follow-up during the postprogram years because they moved from Chicago and could not be located, were deceased, or did not have sufficient identifying information to track, refused to participate, or were incarcerated (other). At 35 years of age, the total number of deceased individuals in the study was 73. Seven individuals who died after July 1, 2009, were included in the study sample. The attrition sample of 141 had missing data on educational attainment between the ages of 28 and 35 years.

### Group Comparability

At follow-up, the program and comparison groups were similar on most characteristics (Table 2). These characteristics were measured from administrative records and family surveys primarily between birth and 5 years of age. The groups were similar in employment, single-parent family status, neighborhood poverty, and adverse experiences (eg, parent substance abuse). They differed in mother’s educational level and child welfare but were equivalent among male participants. The original sample had similar group equivalence (eTable 2 in the Supplement). The original and follow-up samples had similar attributes (eg, family risk, program participation) (eTable 1 in the Supplement). However, the attrition sample had a higher percentage of male participants. Inverse probability weighting accounted for differences in analyses (eTables 3 and 4 in the Supplement).

**Table 2.** Preprogram Attributes of Child-Parent Center Program and Comparison Groups for the Follow-up Study^a^

Child or Family Characteristic	Follow-up Sample at 35 Years of Age (n = 1398)			Male Program vs Control (n = 666)			Female Program vs Control (n = 732)		
	Program Group (n = 904)	Comparison Group (n = 494)	Difference (95% CI)	Program Group (n = 414)	Comparison Group (n = 252)	Difference (95% CI)	Program Group (n = 490)	Comparison Group (n = 242)	Difference (95% CI)
Sample recovery	91.4	89.8	1.6 (–1.5 to 4.7)	87.0	88.5	–1.5 (–6.2 to 3.3)	95.7	93.8	1.9 (–1.5 to 5.3)
Female	54.2	49.0	5.2 (–0.3 to 10.7)						
Black	93.3	93.2	0.1 (–2.6 to 2.9)	93.5	91.3	2.2 (–2.0 to 6.4)	93.1	95.1	–2.0 (–5.5 to 1.6)
Family risk index by child’s age of 3 y^b^	4.49	4.50	–0.7 (–0.19 to 0.18)	4.37	4.52	–0.15 (–0.41 to 0.12)	4.59	4.47	0.12 (–0.14 to 0.37)
≥4 Risk factors by child’s age of 3 y	72.8	70.9	1.9 (–3.0 to 6.9)	69.6	70.3	–0.7 (–7.9 to 6.5)	75.5	71.5	4.0 (–2.8 to 10.9)
Mother did not complete high school by child’s age of 3 y^c^	50.1	58.7	–8.6 (–14.0 to –3.2)^d^	52.7	57.6	–4.9 (–12.7 to 2.9)	48.0	60.0	–12.0 (–19.6 to −4.4)^d^
Mother completed some college by child’s age of 3 y	13.4	10.5	2.9 (–0.6 to 6.4)	13.0	11.5	1.5 (–3.6 to 6.6)	13.7	9.5	4.2 (–0.6 to 9.0)
Single parent by child’s age of 3 y^c^	76.6	75.5	1.0 (–3.7 to 5.7)	74.4	78.2	–3.8 (–10.4 to 2.8)	78.4	72.8	5.6 (–1.1 to 12.3)
Mother not employed by child’s age of 3 y^c^	66.6	62.6	4.0 (–1.2 to 9.3)	61.8	64.7	–2.9 (–10.4 to 4.7)	70.6	60.3	10.3 (2.9 to 17.7)^d^
Ever reported receiving free lunch by child’s age of 3 y^c^	83.6	82.8	0.8 (–3.3 to 5.0)	50.7	52.2	–1.5 (–7.5 to 4.6)	86.1	83.4	2.7 (–2.9 to 8.2)
Ever reported receiving AFDC by child’s age of 3 y^c^	62.5	60.3	2.2 (–3.2 to 7.5)	58.5	61.6	–3.1 (–10.7 to 4.6)	65.9	59.1	6.8 (–0.7 to 14.3)
≥4 Children at home by child’s age of 3 y^c^	16.0	19.0	–3.0 (–7.2 to 1.2)	16.7	15.5	1.2 (–4.5 to 6.9)	15.5	22.7	–7.2 (–13.4 to –1.0)^d^
Children in school area in which ≥60% of children reside in low-income families^c^	77.8	73.1	4.7 (–0.1 to 9.5)	75.6	71.8	3.8 (–3.2 to 10.7)	79.6	74.4	5.2 (–1.3 to 11.8)
Any child welfare history by child’s age of 3 y	2.9	5.5	–2.6 (–4.9 to –0.3)^d^	2.4	5.1	–2.7 (–5.9 to 0.4)	3.3	5.8	–2.5 (–5.9 to 0.8)
Mother was teenager at child’s birth^c^	15.9	17.8	–1.9 (–6.0 to 2.3)	16.7	20.3	–3.6 (–9.7 to 2.6)	15.3	15.3	0 (–5.5 to 5.6)
Missing any family risk indicators	13.7	15.6	–1.9 (–5.8 to 2.0)	15.0	19.1	–4.1 (–10.0 to 1.9)	12.7	12.0	0.7 (–4.4 to 5.7)
Low birth weight (<2500 g)	11.3	14.0	–2.7 (–6.4 to 1.0)	10.6	11.5	–0.9 (–5.8 to 4.1)	11.8	16.5	–4.7 (–10.2 to 0.8)
Home environment problem at ages 0-5 y	51.9	55.7	–3.8 (–9.3 to 1.7)	51.7	55.6	–3.9 (–11.7 to 3.9)	52.0	55.7	–3.7 (–11.0 to 3.9)
No. of adverse childhood experiences at age 0-5 y	0.34	0.31	0.03 (–0.05 to 0.10)	0.42	0.36	0.06 (–0.05 to 0.18)	0.26	0.26	0 (–0.09 to 0.09)

Abbreviation: AFDC, Aid to Families with Dependent Children.

^a^ Data are presented as percentage of individuals unless otherwise indicated. The child and family background indicators were measured from administrative records (birth certificates, school records) primarily from birth to 5 years of age. Parent reports from ages 7 to 12 years were used to supplement some risk indicators (eg, number of children). These and other indicators were used in the inverse probability weighting models. Home environment problems (eg, frequent family conflict) and adverse child experiences were from retrospective reports. The preschool to third grade group showed similar patterns and is not shown.

^b^ The index ranges from 0 to 7, with higher numbers indicating greater levels of family risk.

^c^ Family risk indicators.

^d^ The 95% CI does not include zero.

### Outcomes for Preschool and School-aged Participation

As indicated in Table 3, after controlling for IPW selection and attrition, the preschool group had significantly higher levels of educational attainment on 9 of 12 outcomes. These outcomes included a higher rate of 4-year high school graduation (51.0% vs 44.0%; difference, 7.0%; 95% CI, 1.4%-12.6%), college attendance (61.2% vs 53.1%; difference, 8.1%; 95% CI, 0.8%-15.4%), associate’s degree or higher (15.7% vs 10.7%; difference, 5.0%; 95% CI 1.0%-9.0%), and master’s degree or higher (4.2% vs 1.5%; difference, 2.7%; 95% CI, 1.3%-4.1%) (Figure). The advantage for years of education was a half year (12.81 vs 12.32 years), but the difference for an earned bachelors’ degree was smaller (11.0% vs 7.8%). The only difference for CPC school-age was in on-time high school graduation.

**Table 3.** Adjusted Rates of Educational Attainment by Preschool, School-aged, and Extended-Intervention Group Status^a^

Educational Outcomes by 35 Years of Age	Preschool Groups			School-aged Groups			Extended Intervention Groups^b^		
	Intervention (n = 904)	Comparison (n = 494)	Difference (95% CI)	Intervention (n = 776)	Comparison (n = 622)	Difference (95% CI)	Intervention (n = 514)	Comparison (n = 884)	Difference (95% CI)
Dropout by 16 y of age	11.2	13.9	–2.7 (–6.8 to 1.3)	12.4	11.6	0.8 (–2.2 to 3.8)	11.6	12.6	–1.0 (–5.2 to 3.1)
4-y High school graduation	51.0	44.0	7.0 (1.4 to 12.6)^c^	49.1	46.3	2.8 (–4.4 to 10.0)	55.3	44.7	10.6 (3.4 to 17.8)^c^
On-time high school graduation	42.1	34.3	7.8 (2.5 to 13.1)^c^	42.3	35.1	7.2 (0.8 to 13.6)^c^	48.5	34.7	13.8 (6.0 to 21.6)^c^
High school completion	86.9	80.7	6.2 (0.9 to 11.6)^c^	85.2	83.5	1.7 (–2.5 to 5.9)	87.3	82.8	4.5 (1.3 to 7.8)^c^
High school graduation	56.0	50.5	5.5 (0.3 to 10.8)^c^	53.7	52.4	1.3 (–6.3 to 8.9)	59.6	50.4	9.2 (2.3 to 16.0)^c^
Years of education	12.81	12.32	0.49 (0.20 to 0.77)^c^	12.65	12.55	0.1 (–0.14 to 0.33)	12.95	12.45	0.5 (0.17 to 0.84)^c^
College attendance	61.2	53.1	8.1 (0.8 to 15.4)^c^	59.4	56.5	2.9 (–3.1 to 9.0)	63.2	55.8	7.4 (1.4 to 13.4)^c^
4-y College attendance	29.3	21.4	7.9 (1.9 to 14)	25.6	26.0	–0.4 (–7.1 to 6.4)	31.4	24.0	7.4 (0.4 to 14.4)
Associates’ degree or higher	15.7	10.7	5.0 (1.0 to 9.0)	14.2	13.4	0.8 (–3.8 to 5.3)	18.5	12.5	6.0 (1.0 to 11.0)
Bachelor’s degree or higher	11.0	7.8	3.2 (–0.3 to 6.7)	10.8	8.7	2.1 (–1.8 to 6.0)	14.3	8.2	6.1 (1.3 to 10.9)
Master’s degree or higher	4.2	1.5	2.7 (1.3 to 4.1)	3.8	2.3	1.5 (–0.5 to 3.4)	5.9	2.3	3.6 (1.4 to 5.9)
Postsecondary credential	18.3	17.2	4.1 (–1.1 to 9.3)	20.4	19.2	1.2 (–4.4 to 6.7)	25.0	18.1	6.9 (0.9 to 12.9)

^a^ Data are percentage of individuals unless otherwise indicated. Adjusted with inverse probability weighting for program selection and attrition. Comparisons for other extended intervention groups showed a similar pattern. Child welfare history by 4 years of age was not included in the models of bachelor’s degree and master’s degree or higher because it predicted the outcomes. A total of 57 individuals (4%) reported having a master’s degree or higher. The extended intervention model was estimated separately from the preschool and school-aged model.

^b^ Extended intervention was 4 to 6 years; comparison, less than 4 years.

^c^ The 95% CI does not include zero.

**Figure.**
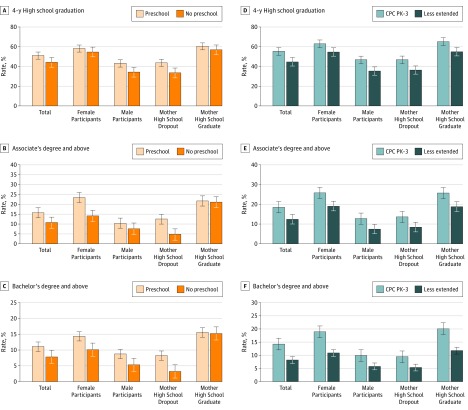
Adjusted Rates for 3 Measures of Educational Attainment by Child-Parent Center (CPC) Program Participation Values are marginal rates adjusted for inverse probability weighting (IPW) for program selection and attrition. A-C, Findings according to preschool CPC participation. In A-C, CPC program school-aged participation was also included in the model (see eTables 3 and 4 in the Supplement for IPW input models and eTables 5-12 in the Supplement for other outcomes). D-F, Findings according to preschool to third grade (PK-3) CPC program participation. Error bars indicate SD.

### Outcomes for Extended Program Participation

Compared with fewer years of participation, the CPC 4- to 6-year group (extended intervention) had higher levels of educational attainment on 11 of 12 outcomes. These outcomes included a higher rate of 4-year high school graduation (55.3% vs 44.7%; difference, 10.6%; 95% CI, 3.4%-17.8%) (Table 3 and Figure), 4-year college attendance (31.4% vs 24.0%; difference, 7.4%; 95% CI, 0.4%-14.4%), associate’s degree or higher (18.5% vs 12.5%; difference, 6.0%; 95% CI, 1.0%-11.0%), bachelor’s degree (14.3% vs 8.2%; difference, 6.1%; 95% CI, 1.3%-10.9%), and postsecondary credential (25.0% vs 18.1%; difference, 6.9%; 95% CI, 0.9%-12.9%) (eFigure 1 in the Supplement). The percentage improvements over the comparison group were 38.1% for associate’s degree or higher and 48.0% for postsecondary credentials. Other comparisons were similar (eTable 5 in the Supplement).

### Duration (Dosage) of Participation to Third Grade

Table 4 gives the levels of attainment for specific dosage groups ranging from 0 to 5 to 6 years of participation. Although, as expected, there were increasing attainment rates as years of intervention increased, 3 results were evident (see biserial correlations and marginal effects for years of intervention in Table 4). First, the largest differences between the minimum and maximum duration of services was for on-time high school graduation, 4-year high school graduation, college attendance, and years of education. For on-time graduation, the difference was 22.6 percentage points (53.2% vs 30.6%; marginal effect, years = 3.0%; 95% CI, 1.8%-4.1%).

**Table 4.** Adjusted Means and Marginal Effects of Select Measures of Educational Attainment by Program Dosage^a^

Measure	Mean Value^b^						Difference (95% CI)	
	PK to Third Grade (n = 160)	PK to Second Grade (n = 351)	PK to First Grade (n = 116)	PK/PK and Kindergarten (n = 277)	No PK/School-aged (n = 149)	No Participation (n = 345)	Marginal Effect, Duration of Intervention	Biserial Correlation
Dropout by 16 y of age	7.9	13.3	9.8	11.6	16.1	13.7	–0.7 (–1.7 to 0.4)	–0.08 (–0.14 to –0.03)
4-y High school graduation	60.7^c^	51.6	42.2	45.0	41.0	43.1	2.1 (0.7 to 3.4)^d^	0.14 (0.09 to 0.20)^d^
On-time high school graduation	53.2^c^	44.4	33.5	35.0	36.8	30.6	3.0 (1.8 to 4.1)^d^	0.18 (0.13 to 0.23)^d^
High school completion	87.8	86.8	87.1	84.9	81.1	79.0	1.6 (0.3 to 2.9)^d^	0.15 (0.09 to 0.20)^d^
High school graduation	65.9^c^	55.2	46.0	49.8	46.3	50.8	1.7 (0.3 to 3.1)^d^	0.11 (0.06 to 0.17)^d^
Years of education	12.89^c^	12.95	12.58	12.59	12.14	12.32	0.10 (0.05 to 0.16)^d^	0.14 (0.09 to 0.19)^d^
College attendance	62.5	61.7	57.7	58.1	53.6	50.7	2.0 (0.6 to 3.4)^d^	0.11 (0.06 to 0.17)^d^
4-y College attendance	27.5	30.6	22.3	28.3	20.0	22.4	1.4 (0.4 to 2.4)^d^	0.12 (0.06 to 0.17)^d^
Associate’s degree or higher	15.5	18.1	11.9	13.6	6.8	9.9	0.9 (0.17 to 1.6)^d^	0.14 (0.09 to 0.19)^d^
Bachelor’s degree or higher	11.0	15.4^c^	9.5	8.5	4.7	7.4	0.8 (0.3 to 1.3)^d^	0.15 (0.10 to 0.20)^d^
Master’s degree or higher	3.1	5.8	4.0	0	1.6	2.2	0.4 (0.3 to 0.6)^d^	0.21 (0.16 to 0.26)^d^
Postsecondary credential	27.3	24.0	15.8	17.7	12.2	17.9	0.9 (–0.1 to 1.8)	0.12 (0.07 to 0.18)^d^

Abbreviation: PK, prekindergarten (preschool).

^a^ Data are presented as percentage of individuals unless otherwise indicated. Adjusted with inverse probability weighting (IPW) for program selection and attrition. The groups define the extent of intervention in years. A total of 57 individuals (4%) reported having a master’s degree or higher. When examined by the 6 groups, the no PK group and the any school-aged group predicted the outcome and thus the rates were not reported. Marginal effect assessed years of Child-Parent Center program participation (range, 0-6 years) after IPW adjustments. Unadjusted biserial correlation between years of intervention (defined by the groups in the table) and the outcome is adjusted to account for dichotomous outcomes.

^b^ PK to third grade represented individuals aged 5 to 6 years; PK to second grade, 4 to 5 years; PK to first grade, 3 to 4 years; PK/PK and kindergarten, 2 to 3 years; no PK/school age, 1 to 3 years; and no participation, 0 years. The reference group for marginal rates is the PK/PK and kindergarten group.

^c^ The 95% CI does not include zero compared with the reference group (PK/PK + kindergarten [2- to 3-year olds]).

^d^ The 95% CI for marginal effects and correlations do not include zero. The exceptions were dropout by 16 years of age and postsecondary credential.

The second result was that the high-dosage groups, which participated in the entire program from preschool to at least second grade (4-5 years or 5-6 years), had the greatest attainment difference with the preschool and kindergarten group for all 3 high school graduation measures and bachelor’s degree or higher (eg, 15.4% vs 8.5% for preschool to second grade vs preschool only). They also had increased educational attainment compared with the lower dosage groups, including the school-aged intervention group with no preschool (eTable 6 in the Supplement).

Third, the attainment levels of the 2 high-dosage groups were similar. Program groups had increases in degree attainment between 28 and 35 years of age that were more than double those of the comparison groups (eFigure 2 in the Supplement).

### Differences by Subgroups

Separate estimates were reported for sex and mothers’ educational level (eTables 7 to 10 in the Supplement). Compared with male participants, female participants had higher rates of educational attainment. Although postsecondary outcomes were increased for female participants, in the extended intervention, the difference was significant for college attendance only (female participants: 73.5% vs 62.6%; difference, 10.9%; 95% CI, 3.3%-18.5%; male participants: 50.8% vs 49.0%; difference, 1.8%; 95% CI, −7.7%-11.2%) (eTable 7 in the Supplement). For mothers’ educational level, differences in years of education, 4-year college attendance, associate’s or higher degree, and postsecondary credential were greater for preschool participants whose mothers dropped out of high school compared with those with mothers who were high school graduates (eTable 9 and eTable 10 in the Supplement). This finding and others for 3 postsecondary outcomes are shown in the Figure. The difference in high school attainment was greatest for male participants, especially as duration of intervention increased (eTable 8 in the Supplement).

### Robustness Analysis

We compared the main IPW model with 3 others: (1) covariate-adjusted model with no IPW, (2) IPW for program selection, and (3) IPW for attrition. As reported in eFigure 4 in the Supplement, program estimates were robust across models that balanced the covariates between groups (eTables 11 and 12 in the Supplement). The covariate-adjusted model yielded slightly lower estimates of impact (eFigures 1, 3, and 4 in the Supplement).

## Discussion

Lower educational attainment is a major risk factor for many indicators of health and well-being. To our knowledge, no previous early childhood studies have investigated degree completion for large-scale programs after the mid-20s. Given the low generalizability and small sample sizes of previous studies,^17,18,22,23^ differences by subgroups and dosage of intervention are underinvestigated. The benefits of continuing services beyond 5 years of age are especially limited.

As the most comprehensive longitudinal study of an established large-scale program, CPC was associated with educational attainment in midlife. We found positive outcomes for 4-year high school graduation, college attendance, and degree completion (associate’s degree or higher). Given that educational attainment is the leading social determinant of health, findings demonstrate that school-based early childhood programs, such as the CPC program, have significant potential to advance life-course health and well-being. For example, all 7 ideal health metrics of the American Heart Association^1,2,3^ (eg, hypertension) are associated with educational attainment in a dosage-response fashion.^36,37^ Higher levels of education are associated with greater economic well-being,^5,38,39^ reduced depression, and involvement in the justice system.^5,6^ Increased access to high-quality programs provides an important avenue of improved well-being.

The program had compensatory effects for those at elevated risk: black male participants and children of school dropouts. Although the results varied by duration of intervention, they indicate that prevention can be particularly effective in increasing educational attainment for those with the largest disparities in outcomes. For example, the preschool group from low-education households had a 2-fold increase in bachelor’s degree attainment compared with the comparison group. This result dovetails with previous findings^14,21,25^ and supports the CPC program in enhancing quality, intensity, and continuity of learning.^27,28,29^

Our focus on 4-year high school graduation is unique and reflects a key Healthy People metric.^9^ The association between intervention and the educational attainment continuum in midlife increases the likelihood that the CPC program can influence adult health.^40^ This finding is supported by increasing literature that early childhood experiences link to healthy behaviors.^39,41^ A major implication is that investments in the ECI, whether during the preschool or early school-aged years, are investments in public health and can affect long-term outcomes. Although access to publicly funded preschool has increased in recent years, only 42% of children aged 4 years and 15% of those aged 3 years enroll in state prekindergarten, Head Start, or other programs.^42^ Continuing participation in programs similar to the CPC program is not tracked but has been estimated to be less than 10%.^22,23,28^ Our findings indicate the importance of increasing the availability of such services.

Another unique feature of the study was examination of preschool to third grade services within a dosage framework. Research on dosage effects have been limited despite their direct policy implications. Studies^43,44^ have found that an additional year of preschool leads to improvements in educational outcomes. Whether the effects are sustained largely depends on the quality of subsequent experiences.^4,23,27^ Our findings extend previous work by showing that years of participation from preschool to second or third grade were linked to all high school graduation outcomes. Years were also associated with higher rates of earned degrees, including a bachelor’s degree. Long-term effects on education can be strengthened if services continue through at least second grade.

The processes that account for the observed links with educational attainment are complex, but several have been identified. Through educational enrichment, the CPC program and other interventions promote early cognitive scholastic skills, which lead to better school performance and adjustment and increased school commitment, thereby reducing the need for treatment.^11,15,17,22^ Similar cumulative processes of effects have been found for family support and parenting behaviors and school support and quality.^22,23,45^ Socioemotional advantages (eg, self-control) also have a key role in explaining links with crime prevention and college persistence.^11,45^ The linchpin of this mediational process is that initial links are sufficiently strong as a function of effective implementation. How this process carries over to physical health and health behaviors in midlife warrants further investigation, but educational attainment is a key conduit that contributes to a process of cumulative advantage.^4,36,37,45^

A rare feature that further strengthens inferences is the availability of complete records of attainment from multiple sources. Education is a continuous process that extends into the 30s for the CLS cohort and in general. The CPC preschool to third graders and preschoolers had nearly doubled rates of degree attainment from 28 to 35 years of age, whereas the rates for comparison groups increased slightly (eFigure 1 in the Supplement). The program groups also had increased rates of earned degrees by nearly 50% (Table 3 and Table 4). Despite economic barriers that make it more difficult for poor and minority youths to pursue postsecondary education,^46^ these findings indicate that prevention early in life can help reduce economic disparities in attainment at a time when the returns to college are increasing.^5,46^

Nevertheless, these sizable benefits do not eliminate disparities across the education continuum with more advantaged groups, especially for degree completion.^47,48^ Early childhood programs, which are targeted to families at higher levels of risk, would not be expected to permanently compensate for continuing disadvantages. For example, the rate of arrest among CLS youths is double the national rate,^49^ and this difference accounts for the higher rates of attainment for female participants in the study and substantially contributes to the lower rates of degree completion compared with the general population.

### Limitations

This study has 3 limitations. First, the findings are based on a quasi-experimental design, and thus the strength of inferences may not be as strong as in well-executed randomized experiments. Nevertheless, the groups were largely equivalent and findings were consistent across analytic methods. Second, measures of postsecondary education were collected from administrative records and supplemented with self-reports. It is possible that some participants were missed from the records search even though we obtained a 91% rate of retention. Third, generalizability of findings may be restricted. Although other programs are supportive of the long-term benefits reported here,^10,11,13^ the population served in our study had relatively high levels of economic disadvantage. In addition, the CPC program has a long history of effectiveness because of its quality and comprehensiveness, and thus results may not apply to programs of modest quality.

## Conclusions

This study suggests that an established and large-scale ECI contributes positively to educational attainment in midlife, a key social determinant of health and well-being. Replication and extension of findings to other contemporary programs, contexts, and samples should further strengthen confidence in the benefits of multiyear and multicomponent preventive interventions.

## Notes

Supplement. **eTable 1.** Study Sample Characteristics by Attrition Status **eTable 2.** Original Study Sample Characteristics by Program Groups and Gender (N = 1539) **eTable 3.** Propensity Score Predictors of Participation in the CPC Program **eTable 4.** Propensity Score Predictors of Being in the Study Sample **eTable 5.** Adjusted Rates and Means for Educational Attainment by Program Groups **eTable 6.** Adjusted Rates, Means, and Marginal Effects for Educational Attainment by Program Dosage **eTable 7.** Educational Attainment by Program Group Status for Females and Males **eTable 8.** Educational Attainment by Gender and Program Dosage **eTable 9.** Educational Attainment by Mothers’ Education and Program Groups **eTable 10.** Educational Attainment by Mothers’ Education and Program Dosage **eTable 11.** Robustness by Analytic Technique for the Program Groups **eTable 12.** Robustness by Analytic Technique and Program Dosage **eMethods.** Study Background, Measures, and Analysis **eFigure 1.** Adjusted Rates for Two Measures of Attainment by Preschool to Third Grade Participation **eFigure 2.** Rate Differences in Educational Attainment Between Ages 28 and 35 by CPC Groups **eFigure 3.** Adjusted Rates for Two Measures of Attainment by Preschool Participation **eFigure 4.** Propensity-Score Weighted Differences by Program Groups
