# Supplementary material for: A Multicomponent, Preschool to Third Grade Preventive Intervention and Educational Attainment at 35 Years of Age
Source: JAMA Pediatr. 2018 Jan 29;172(3):247–56. doi: 10.1001/jamapediatrics.2017.4673 (PMC5885840; doi:10.1001/jamapediatrics.2017.4673)
Supplement: Supplement. — eTable 1. Study Sample Characteristics by Attrition Status eTable 2. Original Study Sample Characteristics by Program Groups and Gender (N = 1539) eTable 3. Propensity Score Predictors of Participation in the CPC Program eTable 4. Propensity Score Predictors of Being in the Study Sample eTable 5. Adjusted Rates and Means for Educational Attainment by Program Groups eTable 6. Adjusted Rates, Means, and Marginal Effects for Educational Attainment by Program Dosage eTable 7. Educational Attainment by Program Group Status for Females and Males eTable 8. Educational Attainment by Gender and Program Dosage eTable 9. Educational Attainment by Mothers’ Education and Program Groups eTable 10. Educational Attainment by Mothers’ Education and Program Dosage eTable 11. Robustness by Analytic Technique for the Program Groups eTable 12. Robustness by Analytic Technique and Program Dosage eMethods. Study Background, Measures, and Analysis eFigure 1. Adjusted Rates for Two Measures of Attainment by Preschool to Third Grade Participation eFigure 2. Rate Differences in Educational Attainment Between Ages 28 and 35 by CPC Groups eFigure 3. Adjusted Rates for Two Measures of Attainment by Preschool Participation eFigure 4. Propensity-Score Weighted Differences by Program Groups [file jamapediatr-172-247-s001.pdf]

Supplementary Online Content

Reynolds A, Ou S-R, Temple JA. A multicomponent, preschool to third grade preventive intervention with educational attainment at 35 years of age. *JAMA Pediatr.* Published online January 29, 2018. doi:10.1001/jamapediatrics.2017.4673

eTable 1. Study Sample Characteristics by Attrition Status

eTable 2. Original Study Sample Characteristics by Program Groups and Gender (N = 1539)

eTable 3. Propensity Score Predictors of Participation in the CPC Program

eTable 4. Propensity Score Predictors of Being in the Study Sample

eTable 5. Adjusted Rates and Means for Educational Attainment by Program Groups

eTable 6. Adjusted Rates, Means, and Marginal Effects for Educational Attainment by Program Dosage

eTable 7. Educational Attainment by Program Group Status for Females and Males

eTable 8. Educational Attainment by Gender and Program Dosage

eTable 9. Educational Attainment by Mothers' Education and Program Groups

eTable 10. Educational Attainment by Mothers' Education and Program Dosage

eTable 11. Robustness by Analytic Technique for the Program Groups

eTable 12. Robustness by Analytic Technique and Program Dosage

eMethods. Study Background, Measures, and Analysis

eFigure 1. Adjusted Rates for Two Measures of Attainment by Preschool to Third Grade Participation

eFigure 2. Rate Differences in Educational Attainment Between Ages 28 and 35 by CPC Groups

eFigure 3. Adjusted Rates for Two Measures of Attainment by Preschool Participation

eFigure 4. Propensity-Score Weighted Differences by Program Groups

This supplementary material has been provided by the authors to give readers additional information about their work.

eTable 1. Study Sample Characteristics by Attrition Status

Characteristics	N	Original sample (n=1539)	Study sample (n=1398)	Attrition sample (n=141)	Mean difference (Study sample-Attrition) (95% CI)
Percent females	1531	50.3	52.4	28.6	23.8 (15.7-31.9)*
Percent Black	1531	92.9	93.2	90.2	3 (-2.2-8.2)
Family risk index (0-7) by child's age 3	1539	4.52	4.49	4.82	-.33 (-0.62-. -0.04)*
Percent four or more risk factors by child's age 3	1531	72.8	72.1	79.7	-7.6 (-14.8-, -0.4)*
Percent mother not completed high school by child's age 3 [1]	1475	54.0	53.4	60.8	-7.5 (-16.6-16.5)
Percent mother completed some college[1]	1475	12.7	12.8	12.5	0.3 (-5.9-6.5)
Percent single parent by child's age 3 [1]	1482	75.6	75.5	76.6	-1.1 (-8.9-6.7)
Percent mother not employed by child's age 3 [1]	1342	62.9	62.2	71.6	-9.4 (-18.6-,-0.2)*
Percent ever reported receiving free lunch by child's age 3 [1]	1445	82.7	82.5	85.2	-2.7 (-9.5-4.1)
Percent ever reported receiving AFDC by child's age 3 [1]	1440	62.2	61.5	69.9	-8.4 (-17.3-0.4)*
Percent having 4 or more children at home by child's age 3[1]	1482	17.3	17.6	13.7	3.9 (-2.5-10.3)
Percentage children in school area in which 60% or more of children reside in low-income families	1531	76.1	76.1	75.9	.2 (-7.4-7.8)
Percent any child welfare case history by child's age 3 [1]	1411	4.1	4.0	4.9	-.9 (-5.2-3.5)
Percent mother was teen at child's birth [1]	1493	16.7	17.0	13.5	3.5 (-2.8-9.8)
Percent missing on any family risk indicators	1531	15.8	14.4	30.8	-16.5 (-24.5-, -8.4)*
Percent low birth weight (<2500 grams)	1456	12.4	12.7	8.6	4.1 (-1.3-9.6)
Percent CPC preschool participation	1539	64.3	64.7	60.3	4.4 (-4.1-12.8)
Percent CPC school-age participation	1539	55.2	55.5	52.5	3.0 (-5.6-11.7)
Percent CPC extended participation	1539	35.9	36.8	27.7	9.1 (1.3-16.9)*

Note. 1. Means reported before imputation for missing data. Characteristics were measured from administrative records (e.g., birth records) from primarily ages 0 to 5. Demographics were measured from school records.

*95% CI does not include zero.

eTable 2. Original Study Sample Characteristics by Program Groups and Gender (N = 1539)

	Original sample			Male P vs C		Female P vs C	
Child or Family Characteristic	Prog. Group (n=989)	Comp. Group (n=550)	Difference (95% CI)	Prog. Group (n=476)	Difference (95% CI)	Prog. Group (n=512)	Difference (95% CI)
Percent females	51.8	47.5	4.3 (-.9-9.5)	--	--	--	--
Percent Black	92.7	93.4	-.7 (-3.3-2)	92.7	1.1 (-2.9-5.1)	92.7	-2.6 (-6-.8)
Family risk index (0-7) by child's age 3	4.52	4.54	-.02 (-.2-.15)	4.42	-.14 (-.39-.11)	4.60	.11 (-.14-.36)
Percent four or more risk factors by child's age 3	73.3	71.8	1.5 (-3-6.1)	70.6	-1 (-7.6-5.7)	75.8	4 (-2.9-10.3)
Percent mother not completed high school by child's age 3[1]	51.0	60.2	-9.2 (-14.4-4.1)**	53.6	-5.0 (-12.3-2.2)	48.4	-12 (-19.8, -5.1)**
Percent mother completed some college child's age 3	13.5	10.0	3.5 (.2-6.7)*	13.7	2.8 (-2-7.5)	13.3	4 (-.6-8.6)
Percent single parent by child's age 3[1]	76.7	75.7	1.0 (-3.4-5.5)	74.8	-3.8 (-10-2.4)	78.5	6 (-.5-12.5)
Percent mother not employed by child's age 3[1]	67.3	64.1	4 (-1.8-8.2)	63.9	-3.2 (-10.1-3.8)	70.5	9.7 (2.5-16.8)**
Percent ever reported receiving free lunch by child's age 3[1]	84.2	82.7	1.5 (-2.4-5.4)	82.4	-.1 (-5.7-5.5)	85.9	3 (-2.5-8.5)
Percent ever reported receiving AFDC by child's age 3[1]	63.1	61.7	1.4 (-3.7-6.4)	59.9	-3.6 (-10.8-3.5)	66.0	6.3 (-.9-13.6)
Percent having 4 or more children at home by child's age 3[1]	16.0	18.1	-2.1 (-6-1.9)	16.0	1.2 (-4-6.5)	16.0	-5.7 (-11.6 -.3)
Percentage children in school area in which 60% or more of children reside in low-income families[1]	77.6	73.3	4.3 (-.2-8.9)	75.6	4.1 (-2.5-10.6)	79.5	4.3 (-2-10.6)
Percent any child welfare case history by child's age 3	3.2	5.0	-1.8 (-4 -.3)	2.9	-1.6 (-4.5-1.2)	3.3	-2.1 (-5.3-1.1)
Percent mother was teen at child's birth[1]	15.6	17.5	-1.9 (-5.8-2)	16.4	-3.3 (-9-2.4)	14.8	-.3 (-5.6-5.1)
Percent missing on any family risk indicators	14.7	17.9	-3.2 (-7.1-.7)	16.0	-5.4 (-11.2-.4)	13.5	-.5 (-5.6-4.7)
Percent low birth weight (<2500 grams)	10.9	13.4	-2.5 (-6-1)	9.7	-1.6 (-6.1-3)	12.1	-3.8 (-9.1-1.5)
Percent home environment problem ages 0-5	51.6	53.8	-2.2 (-7.4-3.1)	51.3	-1 (-8.4-6.3)	52.0	-3.5 (-10.9-4)
Number of adverse childhood experiences, ages 0-5 (range 0-6)	0.34	0.29	.05 (-.02-.11)	0.42	.08 (-.03-.18)	0.26	.02 (-.07-.10)

Note. 1. Family risk indicators. Characteristics were measured from administrative records (e.g., birth records) from primarily ages 0 to 5, parent reports up to age 12, and for home environment problems and adverse child experiences retrospective reports by participants. Demographics were measured from school records. Ns for comparison group males and females were 287 and 256, respectively. 7 comparison group and 1 program group participant had missing gender information.
*95% CI does not include zero.

eTable 3. Propensity Score Predictors of Participation in the CPC Program

	Preschool		School-age		Extended		No CPC[1]		CPC extension[1]	
Predictors	Coef.	P>z	Coef.	P>z	Coef.	P>z	Coef.	P>z	Coef.	P>z
Constant term	2.124	0.087	-0.001	0.999	-0.038	0.962	-0.484	0.794	-0.838	0.577
Mother did not complete HS, child age 0-3	-0.288	0.003	-0.168	0.040	-0.256	0.003	-0.019	0.017	0.015	0.062
Child eligible for subsidized meals, child age 0-3	0.048	0.631	-0.026	0.812	-0.032	0.806	0.191	0.063	-0.142	0.165
Mother under age 18 at child birth	-0.056	0.367	0.031	0.795	-0.055	0.621	-0.179	0.201	-0.123	0.544
Four or more children in family, child age 0-3	-0.168	0.069	-0.056	0.570	-0.045	0.588	0.109	0.442	0.042	0.842
Participate in AFDC program, child age 0-3	-0.051	0.550	0.100	0.156	0.110	0.199	0.171	0.359	0.080	0.656
Mother not employed, child age 0-3	0.148	0.111	0.032	0.743	-0.003	0.978	0.117	0.300	0.250	0.036
Single parent family status, child age 0-3	-0.001	0.988	-0.027	0.747	0.010	0.897	-0.294	0.052	-0.160	0.302
Indicator for missing risk factors, child age 0-3	-0.148	0.068	-0.351	0.000	-0.470	0.000	0.081	0.450	0.057	0.594
Reside in high poverty neighborhood	1.679	0.000	0.356	0.415	0.467	0.255	-0.133	0.334	-0.743	0.000
Low birth weight (<2500g)	-0.254	0.002	-0.091	0.486	-0.244	0.029	-2.431	0.000	-0.890	0.135
Child underage at preschool entry	-0.148	0.160	-0.112	0.267	-0.109	0.335	0.217	0.213	-0.111	0.574
Family conflict, child age 0-5	0.020	0.878	0.138	0.213	0.120	0.357	-0.011	0.956	-0.146	0.526
Family financial problems, child age 0-5	-0.002	0.987	-0.044	0.709	-0.285	0.003	0.394	0.108	0.366	0.116
Substance abuse parent, child age 0-5	0.108	0.417	-0.109	0.487	0.196	0.121	-0.040	0.869	-0.419	0.024
Female child	0.104	0.058	-0.002	0.982	0.083	0.283	-0.359	0.129	0.034	0.877
African American child	-0.247	0.686	0.018	0.966	-0.125	0.714	-0.011	0.919	0.036	0.763
Age in months at kindergarten	-0.033	0.028	0.002	0.896	-0.004	0.773	0.207	0.722	-0.125	0.392

Note: N = 1,531. Estimates from probit regression were use in the Inverse Propensity Score models. 1. Coefficients are from a multinomial probit regression. Base outcome is no CPC extended intervention group. The extended intervention group had 4 to 6 years of CPC (preschool to 2nd or 3rd grade). AFDC = Aid to Families with Dependent Children.

eTable 4. Propensity Score Predictors of Being in the Study Sample

	Coefficient	95% CI	P-value
Any CPC preschool	.064	-.091 - .219	.418
Any CPC school age	-.110	-.329 - .110	.328
ITBS Kindergarten word analysis score	.002	-.006 - .009	.586
Mother did not complete HS, child age 0-3	-.218	-.386 - -.051	.011
Child eligible for subsidized meals, child age 0-3	.027	-.258 - .312	.852
Mother under age 18 at child birth	.312	.105 - .520	.003
Four or more children in family, child age 0-3	.196	-.106 - .497	.203
Participate in AFDC program, child age 0-3	-.108	-.354 - .138	.391
Mother not employed, child age 0-3	-.165	-.427 - .098	.218
Single parent family status, child age 0-3	-.049	-.299 - .201	.700
Indicator for missing risk factors, child age 0-3	-.279	-.508 - -.050	.017
Reside in high poverty neighborhood	-.070	-.276 - .137	.508
Low birth weight (<2500g)	.265	-.053 - .583	.102
Family conflict, child age 0-5	.246	-.147 - .640	.220
Family financial problems, child age 0-5	.641	.178 - 1.104	.007
Substance abuse parent, child age 0-5	-.142	-.683 - .398	.606
Female child	.510	.311 - .709	.000
African American child	.145	-.114 - .405	.273
Early residential mobility	-.087	-.197 - .022	.119
Have a SSN	.676	.301 - 1.051	.000
Percent Living one year in current unit, Neighborhood census	-2.049	-5.533 - 1.434	.249
Percent living 1-5 years in current unit, Neighborhood census	-.380	-3.373 - 2.612	.803
Percent living 5-10 years in current unit, Neighborhood census	-.511	-3.948 - 2.925	.771
Percent living 10-20 years in current unit, Neighborhood census	-1.285	-4.526 - 1.957	.437
Percent Self-employed, neighborhood census	-2.564	-5.574 - .446	.095
Percent Black families with female householder, neighborhood census	-.894	-1.423 - -.365	.001

Note. Original sample = 1,531. Educational attainment study sample is 1,398. Standard errors are clustered at the site level. We estimate the probability of being in the retained sample using a probit regression model, controlling for child characteristics, family risk factors, neighborhood variables and having permission to track them using their social security number, and report the marginal effects.

eTable 5. Adjusted Rates and Means for Educational Attainment by Program Groups

	Preschool Groups		School-age Groups		Extended Group-1		Extended Group-2		Extended Group-3	
Education Outcomes by Age 35	Interv n=904	Comp n=494	Interv n=776	Comp n=622	Interv (4-6 years) n=514	Comp (less than 4 years) n=884	Interv (4-6 years) n=514	Comp (0 year) n=345	Interv (4-6 years) N=514	Comp (Pk+K) N=390
Dropout by age 16. %	10.8	14.4	12.4	11.4	11.3	12.4	10.6	12.9	12.6	11.6
4-year high school graduation, %	51.7*	45.2	50.6	47.6	56*	45.6	56.2*	45.4	55.0*	46.1
On-time high school graduation, %	42.7*	36.4	44.0*	36.2	49.3*	35.5	51*	34.6	48.5*	36.0
High school completion, %	87.0*	81.0	86.0	83.9	87.9*	83.1	89.3*	82.1	86.1	84.5
High school graduation, %	56.3	51.1	55.3	53.5	60.3*	51.1	59.9*	51.4	59.9*	51.3
Year of education (range 7-22)	12.81*	12.36	12.69	12.59	12.96*	12.47	13.02*	12.44	12.85*	12.53
College attendance, %	61.2*	54.4	60.1	57.1	63.2*	56.2	65.3*	54.8	61.2	57.4
4-year college attendance, %	29.1*	21.9	26.5	26.7	31.0*	24.2	32.3*	23.5	29.1	25.2
Certificate, %	5.5	5.4	5.4	5.4	6.3	4.9	5.5	5.2	6.8	4.7
Associate degree, %	6.3	4.1	4.7	6.7	5.4	5.8	5.9	5.4	4.6	6.2
Associates' degree or higher, %	16.4*	11.4	14.8	14.1	18.3*	12.6	18.8*	12.3	16.9	13.1
Bachelors' degree or higher, %	11.5	8.2	11.3	9.1	14.1*	8.3	14.1*	8.3	13.6*	8.5
Masters' degree or higher, %	4.2*	1.9	3.8	2.5	5.8*	2.2	5.5*	2.5	5.0*	2.4
Postsecondary Credential, %	21.8	17.9	20.8	19.8	24.3*	18.2	23.9	18.4	23.8	18.4

Note. Adjusted with IPW attrition and selection. Gender, race, and sociodemographic factors are included as covariates. Child welfare history by age 4 is not included in the models of Bachelors' degree and Masters' degree or higher because it predicted the outcomes perfectly. There are 57 cases (4%) report Masters' degree or higher.
*95% CI does not include zero.

eTable 6. Adjusted Rates, Means, and Marginal Effects for Educational Attainment by Program Dosage

Education Outcomes by Age 35	Group 1	Difference (95% CI)	Group 2	Difference (95% CI)	Group 3	Difference (95% CI)	Group 4[1]	Group 5	Difference (95% CI)	Group 6	Difference (95% CI)	Correlation (95% CI)
Dropout by age 16, %	7.9	-3.7 (-10.5 to 3.2)	13.3	1.7 (-3.0 to 6.4)	9.8	-1.8 (-9.3 to 5.7)	11.6	16.1	4.5 (-2.1 to 11.1)	13.7	2.1 (-3.9 to 8.1)	-.08 (-.14, -.03)
4-year high school graduation, %	60.7*	15.7 (3.6 to 27.8)	51.6	6.6 (-3.2 to 16.4)	42.2	-2.8 (-16.9 to 11.3)	45.0	41.0	-4.1 (-12.3 to 4.2)	43.1	-1.9 (-10.9 to 7.2)	.14 (.09 to .20)
On time high school graduation, %	53.2*	18.2 (4.2 to 32.2)	44.4	9.4 (-1.3 to 20.1)	33.5	-1.5 (-13.4 to 10.4)	35.0	36.8	1.8 (-5.7 to 9.4)	30.6	-4.4 (-14.8 to 5.9)	.18 (.13 to .23)
High school completion, %	87.8	2.9 (-2.5 to 8.3)	86.8	1.9 (-3.6 to 7.5)	87.1	2.2 (-4.8 to 9.3)	84.9	81.1	-3.8 (-13.6 to 6.1)	79.0	-5.9 (-12.2 to 0.3)	.15 (.09 to .20)
High school graduation, %	65.9**	16.1 (4.4 to 27.9)	55.2	5.4 (-4.5 to 15.4)	46.0	-3.8 (-18.5 to 10.9)	49.8	46.3	-3.5 (-12.5 to 5.5)	50.8	1.0 (-9.8 to 11.8)	.11 (.06 to .17)
Year of education (range 7-22)	12.89*	.30 (.07 to .53)	12.95	.36 (-.08 to .80)	12.58	-.01 (-.41 to .39)	12.59	12.14*	-.45 (-.75 to -.15)	12.32	-.27 (-.71 to .18)	.14 (.09 to .19)
Any college attendance, %	62.5	4.4 (-3.6 to 12.5)	61.7	3.6 (-5.2 to 12.5)	57.7	-0.4 (-12.6 to 11.7)	58.1	53.6	-4.5 (-17.0 to 8.0)	50.7	-7.4 (-19.9 to 5.2)	.11 (.06 to .17)
4-year college attendance, %	27.5	-0.8 (-6.1 to 4.5)	30.6	2.3 (-8.6 to 13.1)	22.3	-6.0 (-16.1 to 4.1)	28.3	20.0*	-8.3 (-16.5 to -0.2)	22.4	-5.9 (-14.4 to 5.6)	.12 (.06 to .17)
Associates' degree or higher, %	15.5	1.9 (-3.5 to 7.2)	18.1	4.5 (-3.5 to 12.4)	11.9	-1.7 (-8.4 to 5.0)	13.6	6.8*	-6.8 (-11.8 to -1.8)	9.9	-3.7 (-9.9 to 2.5)	.14 (.09 to .19)
Bachelors' degree or higher, %	11.0	2.5 (-0.4 to 5.3)	15.4*	6.9 (-0.2 to 14)	9.5	1.0 (-5.0 to 7.0)	8.5	4.7	-3.8 (-7.3 to -0.3)	7.4	-1.1 (-5.6 to 3.5)	.15 (.10 to .20)
Postsecondary Credential, %	27.3*	9.6 (4.4 to 14.8)	24.0	6.3 (-3.2 to 15.7)	15.8	-1.9 (-11.2 to 7.5)	17.7	12.2	-5.5 (-12.1 to 1.1)	17.9	0.2 (-8.3 to 8.8)	.12 (.07 to .18)

Note. Adjusted with IPW attrition and selection. Gender, race, and sociodemographic factors are included as covariates. Child welfare history by age 4 is not included in the models of Bachelor s' degree and Masters' degree or higher because it predicted the outcomes perfectly. There are 57 cases (4%) report Master degree or higher. When examined by the 6 groups, the "no prek had any follow on group" predicted the outcome perfectly, so it was not examined. The correlation is the unadjusted biserial correlation between the extent of intervention in years and the outcome, and it adjusts for the dichotomous outcomes.

Groups are as follows: (1)Prek to 3rd grade, n=160 (2) Prek to 2nd grade n=351 (3) Prek to 1st grade n=116 (4) Prek/Prek + K n=277[1] (5) No Prek had anyfollow on n=149 (6) No participation n=345.

[1]The reference group for all comparisons and 95% CIs is the Prek/Prek + K group. *95% CI does not include zero.

eTable 7. Educational Attainment by Program Group Status for Females and Males

	Preschool Groups		School-age groups		Extended Group-1		Extended Group-2		Extended Group-3	
Education Outcomes by Age 35	Interv	Comp	Interv	Comp	Interv (4-6 years)	Comp (less than 4 years	Interv (4-6 years)	Comp (0 year)	Interv (4-6 years)	Comp (Pk+K)
**Female (N=732)**	**n=490**	**n=242**	**n=409**	**n=323**	**n=282**	**n=450**	**n=282**	**n=176**	**n=282**	**n=208**
4-year high school graduation, %	58.2	54.6	59.6	53.6	62.9	54.7	64.0*	53.6	62.6	55.1
On-time high school graduation, %	49.2	46.5	54.3*	41.7	58.4*	43.5	59.8*	42.2	58.7*	43.2
High school completion, %	90.7	89.8	92.2*	87.5	92.6*	88.6	93.0*	88.1	92.6	88.7
High school graduation, %	63.1	62.7	66.0	59.2	68.5*	60.8	68.8*	60.5	69.1	60.1
Year of education	13.14+	12.76	13.14	12.93	13.42*	12.86	13.39	12.89	13.39*	12.88
College attendance, %	69.4*	58.8	67.4	65.1	73.5*	62.6	74.8*	61.0	71.7	64.5
4-year college attendance, %	35.6+	26.9	32.1	34.3	38.1	30.5	37.9	30.7	37.4	31.2
Associates' degree or higher, %	23.4*	14.1	20.7	21.2	25.8	19.1	25.7	19.0	24.2	20.2
Bachelors' degree or higher, %	14.3	10.1	15.5	11.6	19.0*	10.9	17.5	12.0	19.4*	10.6
Masters' degree or higher, %	5.7+	2.7	5.4	3.7	7.0*	3.6	5.9	4.3	7.0	3.7
Postsecondary credential, %	29.3	22.1	28.2	26.1	32.5	25.2	31.6	26.0	31.7	25.9
**Male (N=666)**	**n=414**	**n=252**	**n=367**	**n=299**	**n=232**	**n=434**	**n=232**	**n=169**	**n=232**	**n=182**
4-year high school graduation, %	43.1	34.1	39.0	38.4	46.7*	35.4	46.7	35.4	45.5	36.4
On-time high school graduation, %	34.0*	24.5	31.1	27.9	37.9*	26.3	39.3*	25.0	36.6	27.3
High school completion, %	83.8*	71.1	77.1	80.2	81.2	77.0	83.7*	74.2	77.3	81.6
High school graduation, %	47.9	38.9	42.0	44.4	48.9	40.7	48.8	40.9	47.4	42.2
Year of education	12.42*	11.83	12.15	12.14	12.45*	12.04	12.57*	11.92	12.31	12.18
College attendance, %	50.8	48.0	50.0	47.7	50.8	49.0	52.9	46.9	49.9	50.0
4-year college attendance, %	23.6*	16.2	19.8	18.9	24.3	18.4	26.1	16.8	22.4	20.0
Associates' degree or higher, %	10.4	7.6	9.7	7.4	12.8*	7.5	13.9*	6.8	12.5*	7.7
Bachelors' degree or higher, %	8.7	5.3	7.1	6.0	9.9	5.8	10.9*	5.1	9.2	6.3
Masters' degree or higher, %	2.2*	0.2	1.8*	0.4	4.0*	0.6	5.1*	0.4	2.6*	0.7
Postsecondary credential, %	15.0	12.8	13.9	12.8	18.7*	12.2	18.9	12.0	18.9	12.0

Note. Adjusted with IPW attrition and selection. Race, and sociodemographic factors are included as covariates. Child welfare history by age 4 is not included in the models of Bachelors' degree or higher and Masters' degree or higher because it predicted the outcomes perfectly. *95% CI does not include zero.

eTable 8. Educational Attainment by Gender and Program Dosage

Educational attainment	Prek to 3rd grade	Prek to 2nd grade	Prek to 1st grade	Prek only or Prek +K[1]	No Prek had any follow on	No participation
**Female (N=732)**	**n=92**	**n=189**	**n=62**	**n=147**	**n=66**	**n=176**
4-year high school graduation, %	60.6	61.1	53.2	52.4	65.0	48.6
On-time high school graduation, %	58.2*	54.1	41.8	40.5	61.1*	38.3
High school completion, %	92.6	93.4*	91.7	87.7	92.9	86.5
High school graduation, %	71.8*	64.8	55.4	56.7	75.5	56.1
Year of education	13.25*	13.44*	12.97	12.86	12.61	12.80
College attendance, %	75.3*	73.4	61.4	64.6	61.0	57.7
4-year college attendance, %	38.1	37.6	23.3	34.5	26.6	28.9
Associates' degree or higher, %	29.6	24.2	20.4	20.4	6.7*	16.4
Bachelors' degree or higher, %	15.7*	21.1*	13.0	9.2	5.2	11.2
Masters' degree or higher, %	--	--	--	--	--	--
Postsecondary credential, %	38.1**	30.6	27.6	23.7	15.7	24.2
**Male (N=666)**	**n=68**	**n=162**	**n=54**	**n=130**	**n=83**	**n=169**
4-year high school graduation, %	57.9*	42.7	34.4	37.2	28.5	35.9
On-time high school graduation, %	43.4	35.6	25.6	29.6	24.1	22.0
High school completion, %	85.5	80.4	84.6	82.8	72.1	70.6*
High school graduation, %	58.1	46.0	39.7	42.4	31.1	43.0
Year of education	12.54	12.39	12.21	12.26	11.75*	11.78
College attendance, %	49.1	48.6	53.4	47.7	49.7	44.0
4-year college attendance, %	18.2	24.2	21.2	21.2	12.6*	16.1
Associates' degree or higher, %	8.0	12.1	6.6	8.6	6.2	5.3
Bachelors' degree or higher, %	6.9	9.6	6.7	7.1	3.3	4.0
Masters' degree or higher, %	--	--	--	--	--	--
Postsecondary credential, %	20.8*	16.6	6.4	12.3	9.7	11.9

Note. Adjusted with IPW attrition and selection. Race, and sociodemographic factors are included as covariates.
Child welfare history by age 4 is not included in the models of Associates' degree or higher (4 groups only), Bachelors' degree or higher and masters' degree or higher because it predicted the outcomes perfectly.
Masters' degree values could not estimated by gender du to small sample sizes.
1. Reference group. *95% CI does not include zero.

eTable 9. Educational Attainment by Mothers' Education and Program Groups

	Preschool Groups		School-age groups		Extended Group		Extended Group-1		Extended Group-2	
Education Outcomes by Age 35	Interv	Comp	Interv	Comp	Interv (4-6 years)	Comp (less than 4 years)	Interv (4-6 years)	Comp (0 year)	Interv (4-6 years)	Comp (Pk+K)
**Mother completed HS (N=655)**	**n=451**	**n=204**	**n=379**	**n=276**	**n=268**	**n=387**	**n=268**	**n=145**	**n=268**	**n=183**
Dropout by age 16, %	9.7	9.5	10.0	9.3	11.1	8.8	9.9	9.6	11.8	8.1
4-year high school graduation, %	60.3	56.8	61.2	55.6	65.1*	54.9	66.1	53.9	65.2*	54.8
On-time high school graduation, %	52.1	45.3	54.1	44.6	58.7*	44.2	60.5*	42.4	58.8*	44.2
High school completion, %	90.4	89.2	90.2	88.4	91.1	88.2	91.4	87.9	91.0	88.3
High school graduation, %	66.2	61.8	64.7	63.2	68.8	61.2	69.0	60.8	68.7	61.2
Year of education	13.16	13.02	13.12	12.96	13.30	12.89	13.25	12.94	13.33	12.87
4-year college attendance, %	33.9	33.8	34.6	31.5	37.2	31.0	36.4	31.6	38.3	30.0
College attendance, %	67.7	65.9	68.8	64.8	70.7	65.2	70.7	65.1	71.4	64.4
Associates' degree or higher, %	21.7	21.1	23.3	18.7	25.7	18.8	24.9	19.4	26.3	18.3
Bachelors' degree or higher, %	15.5	15.2	16.7	12.4	20.1*	11.8	18.5	12.9	21.3*	11.0
Masters' degree or higher, %	5.7*	2.7	5.5	3.1	7.7*	3.0	6.3	3.6	7.7*	3.0
Postsecondary credential, %	27.0	28.9	29.0	25.1	31.3	25.1	28.5	27.6	33.4*	23.1
**Mother not completed HS (N=743)**	**n=453**	**n=290**	**n=397**	**n=346**	**n=246**	**n=497**	**n=268**	**n=200**	**n=268**	**n=207**
Dropout by age 16, %	12.4	18	14.2	13.5	11.6	16.1	11.2	16.6	13.1	14.3
4-year high school graduation, %	43.6*	33.4	39.0	38.7	46.7*	36.5	46.7*	36.5	44.8	38.2
On-time high school graduation, %	34.1*	25.6	33.0	27.6	39.6*	27.3	40.8*	26.1	38.0	28.5
High school completion, %	83.4*	71.4	80.0	78.5	83.7*	77.2	85.6*	75.0	80.7	81.1
High school graduation, %	47.9*	40.4	44.3	43.6	51.4*	41.5	50.9*	42.0	50.3	42.4
Year of education	12.52*	11.70	12.26	12.18	12.67*	12.06	12.78*	11.93	12.47	12.25
4-year college attendance, %	25.9*	12.5	19.2	21.1	26.5*	18.4	29.2*	16.2	22.6	21.4
College attendance, %	55.7*	42.2	52.1	49.2	56.8*	47.9	59.9*	44.8	53.2	51.8
Associates' degree or higher, %	12.5*	4.7	8.6	9.9	13.6*	8.5	15.4*	7.3	11.2	10.0
Bachelors' degree or higher, %	8.2*	3.2	6.7	5.8	9.5*	5.4	10.8*	4.6	7.8	6.4
Masters' degree or higher, %	2.8*	0.6	2.0	1.3	3.7*	1.3	4.3*	1.1	2.9	1.6
Postsecondary credential, %	17.7*	9.6	14.7	14.8	20.5*	13.3	22.1*	12.0	18.0	15.0

Note. Adjusted with IPW attrition and selection. Gender, race, and sociodemographic factors are included as covariates. Child welfare history by age 4 is not included in the models of certificate, AA degree, and postsecondary education. Race is not included in the model of high school completion. Mather age by age 3 is not included in the model of Masters' degree or higher. Those covariates were not included in those models because they predicted the outcomes perfectly. *95% CI does not include zero.

eTable 10. Educational Attainment by Mothers' Education and Program Dosage

Educational attainment	Prek to 3rd grade	Prek to 2nd grade	Prek to 1st grade	Prek only or Prek +K[1]	No Prek had any follow on	No participation
**Mother completed HS (N=655)**	**n=95**	**n=173**	**n=52**	**n=131**	**n=59**	**n=145**
Dropout by age 16, %	7.9	13.4	6.0	10.0	7.1	10.3
4-year high school graduation, %	71.4*	60.6	49.6	54.7	61.8	53.0
On-time high school graduation, %	67.6*	52.8	39.0	45.1	55.6	39.2
High school completion, %	92.5	90.4	86.6	89.3	88.7	87.3
High school graduation, %	79.7*	61.7	53.5	61.7	64.9	60.7
Year of education	13.36	13.24	12.74	12.91	12.67	13.01
4-year college attendance, %	34.4	36.0	21.4	30.3	32.5	33.8
College attendance, %	67.2	68.4	60.0	62.3	68.3	63.6
Associates' degree or higher, %	24.4	24.2	17.5	17.2	14.1	18.2
Bachelors' degree or higher, %	18.1	21.0	9.3	11.5	8.7	13.3
Masters' degree or higher, %	-	-	-	-	-	-
Postsecondary credential, %	35.0*	27.7	19.6	20.7	18.9	29.5
**Mother not completed HS (N=743)**	**n=65**	**n=178**	**n=64**	**n=146**	**n=90**	**n=200**
Dropout by age 16, %	7.7	13.0	18.4	12.5	23.8*	16.5
4-year high school graduation, %	73.0	52.1	37.6	37.7	1.4	30.7
On-time high school graduation, %	40.0	37.4	29.9	27.6	22.6	23.6
High school completion, %	83.5	83.0	87.9	81.3	73.3	70.9*
High school graduation, %	53.6	48.6	41.4	40.7	31.3	42.4
Year of education	12.51	12.69	12.49	12.33	11.68*	11.72*
4-year college attendance, %	21.3	26.2	23.4	26.5	11.5*	13.6*
College attendance, %	58.9	56.1	57.4	55.1	41.4	39.9*
Associates' degree or higher, %	9.4	14.3	9.1	11.9	3.1*	4.7*
Bachelors' degree or higher, %	5.2	11.3	10.0	6.6	2.2*	3.3
Masters' degree or higher, %	-	-	-	-	-	-
Postsecondary credential, %	21.6	21.4	14.1	16.0	7.9*	9.3

Note. Adjusted with IPW attrition and selection. Race, and sociodemographic factors are included as covariates. Child welfare history by age 4 is not included in the models of Associates' degree or higher (4 groups only), Bachelors' degree or higher and Masters' degree or higher because it predicted the outcomes perfectly. Masters' degree values could not be estimated due to small sample sizes.
1. Reference group for all comparisons and 95% CIs. *95% CI does not include zero.

eTable 11. Robustness by Analytic Technique for the Program Groups

	Preschool Groups		School-age groups		Extended Group		Extended Group-1		Extended Group-2	
Education Outcomes by Age 35	Interv n=904	Comp n=494	Interv n=776	Comp n=622	Interv (4-6 years) n=514	Comp (less than 4 years) n=884	Interv (4-6 years) n=514	Comp (0 year) n=345	Interv (4-6 years) N=514	Comp (Pk+K) N=390
**Dropout by age 16, %**										
Adjusted with covariates	10.8	14.4	12.4	11.4	11.3	12.4	10.6	12.9	12.6	11.6
Adjusted with attrition correction	10.8	14.6	12.5	11.6	11.4	12.5	10.5	13.0	12.9	11.6
Adjusted with selection correction	11.1	13.7	12.4	11.5	11.4	12.5	10.7	13.3	12.6	11.3
Adjusted with attrition & selection	11.2	13.9	12.4	11.6	11.6	12.6	10.8	13.5	12.9	11.3
**4-year high school graduation, %**										
Adjusted with covariates	51.7*	45.2	50.6	47.6	56*	45.6	56.2*	45.4	55.0*	46.1
Adjusted with attrition correction	51.2*	43.9	49.6	47.4	55.3*	44.9	55.6*	44.7	54.0*	45.5
Adjusted with selection correction	51.4*	45.1	50.3	46.7	56.0*	45.3	56.3*	44.9	55.4*	45.8
Adjusted with attrition & selection	51.0*	44.0	49.1	46.3	55.3*	44.7	55.7*	44.3	54.6*	45.4
**On time high school graduation, %**										
Adjusted with covariates	42.7*	36.4	44.0*	36.2	49.3*	35.5	51*	34.6	48.5*	36.0
Adjusted with attrition correction	42.3*	35.2	42.7*	35.9	48.5*	34.9	50.3*	34.0	47.3*	35.5
Adjusted with selection correction	42.5*	35.5	43.6*	35.4	49.3*	35.2	50.7*	33.9	48.8*	35.6
Adjusted with attrition & selection	42.1*	34.3	42.3*	35.1	48.5*	34.7	49.8*	33.3	47.8*	35.2
**High school completion, %**										
Adjusted with covariates	87.0*	81.0	86.0	83.9	87.9*	83.1	89.3*	82.1	86.1	84.5
Adjusted with attrition correction	86.9*	80.3	85.4	83.9	87.5*	82.9	89.0*	81.8	85.4	84.4
Adjusted with selection correction	87.0*	81.4	85.8	83.5	87.7*	83.0	88.7*	81.6	86.4	84.5
Adjusted with attrition & selection	86.9*	80.7	85.2	83.5	87.3*	82.8	88.4*	81.4	85.9	84.5
**High school graduation, %**										
Adjusted with covariates	56.3	51.1	55.3	53.5	60.3*	51.1	59.9*	51.4	59.9*	51.3
Adjusted with attrition correction	56.0*	49.8	54.2	53.3	59.7*	50.5	59.3*	50.8	59.0*	50.9
Adjusted with selection correction	56.3	51.5	54.9	52.6	60.9*	50.9	60.0*	51.1	60.1*	51.1
Adjusted with attrition & selection	56.0*	50.5	53.7	52.4	59.6*	50.4	59.4*	50.6	59.1*	50.7
**Year of education (range 7-22)**										
Adjusted with covariates	12.81*	12.36	12.69	12.59	12.96*	12.47	13.02*	12.44	12.85*	12.53
Adjusted with attrition correction	12.79*	12.32	12.66	12.58	12.93*	12.45	12.99*	12.41	12.80	12.51
Adjusted with selection correction	12.83*	12.36	12.70	12.57	12.98*	12.47	13.02*	12.43	12.90	12.54
Adjusted with attrition & selection	12.81*	12.32	12.65	12.55	12.95*	12.45	12.99*	12.41	12.87	12.53
**College attendance, %**										

	Preschool Groups		School-age groups		Extended Group		Extended Group-1		Extended Group-2	
Education Outcomes by Age 35	Interv n=904	Comp n=494	Interv n=776	Comp n=622	Interv (4-6 years) n=514	Comp (less than 4 years) n=884	Interv (4-6 years) n=514	Comp (0 year) n=345	Interv (4-6 years) N=514	Comp (Pk+K) N=390
Adjusted with covariates	61.2*	54.4	60.1	57.1	63.2*	56.2	65.3*	54.8	61.2	57.4
Adjusted with attrition correction	60.9*	53.8	59.5	56.9	62.6*	55.9	64.9*	54.6	60.4	57.2
Adjusted with selection correction	61.6*	53.7	60.1	56.8	63.8*	56.0	65.4*	54.4	62.6	57.5
Adjusted with attrition & selection	61.2*	53.1	59.4	56.5	63.2*	55.8	64.9*	54.1	61.8	57.3
**4-year college attendance, %**										
Adjusted with covariates	29.1*	21.9	26.5	26.7	31.0*	24.2	32.3*	23.5	29.1	25.2
Adjusted with attrition correction	29.2*	21.3	25.8	26.6	30.6	23.9	32.0	23.2	28.4	24.9
Adjusted with selection correction	29.6*	22.0	26.3	26.1	31.8*	24.3	32.6	23.5	30.5	25.4
Adjusted with attrition & selection	29.3*	21.4	25.6	26.0	31.4*	24.0	32.2	23.1	29.9	25.2
**Associates' degree or higher, %**										
Adjusted with covariates	16.4*	11.4	14.8	14.1	18.3*	12.6	18.8*	12.3	16.9	13.1
Adjusted with attrition correction	16.0*	10.8	14.3	13.7	17.7*	12.2	18.6*	11.9	16.3	12.8
Adjusted with selection correction	16.2*	11.2	14.7	13.8	19.0*	12.8	19.3	12.6	18.0	13.5
Adjusted with attrition & selection	15.7*	10.7	14.2	13.4	18.5*	12.5	18.9*	12.1	17.5	13.2
**Bachelors' degree or higher, %**										
Adjusted with covariates	11.5	8.2	11.3	9.1	14.1*	8.3	14.1*	8.3	13.6*	8.5
Adjusted with attrition correction	11.2*	7.8	10.8	8.9	13.8*	8.0	13.8*	8.0	13.1	8.3
Adjusted with selection correction	11.3	8.1	11.2	8.9	14.7*	8.4	14.3	8.5	14.3*	8.6
Adjusted with attrition & selection	11.0	7.8	10.8	8.7	14.3*	8.2	14.1*	8.3	13.9*	8.4
**Masters' degree or higher, %**										
Adjusted with covariates	4.2*	1.9	3.8	2.5	5.8*	2.2	5.5*	2.5	5.0*	2.4
Adjusted with attrition correction	4.1*	1.8	3.7	2.5	5.5*	2.1	5.3*	2.2	4.7*	2.3
Adjusted with selection correction	4.3*	1.6	4.0	2.4	6.2*	2.4	5.7*	2.5	5.5*	2.6
Adjusted with attrition & selection	4.2*	1.5	3.8	2.3	5.9*	2.3	5.6*	2.4	5.3*	2.5
**Postsecondary Credential, %**										
Adjusted with covariates	21.8	17.9	20.8	19.8	24.3*	18.2	23.9	18.4	23.8	18.4
Adjusted with attrition correction	21.4	17.5	20.3	19.5	23.9*	17.8	23.5	18.1	23.5	18.1
Adjusted with selection correction	21.7	17.6	20.9	19.5	25.3*	18.4	24.8	18.9	25.2	18.6
Adjusted with attrition & selection	18.3	17.2	20.4	19.2	25.0*	18.1	24.4	18.5	24.7	18.2

Note. Gender, race, and sociodemographic factors are included as covariates. Child welfare history by age 4 is not included in the models of Bachelors' degree and Masters' degree or higher because it predicted the outcomes perfectly. There are 57 cases (4%) report Masters' degree or higher. *95% CI does not include zero.

eTable 12. Robustness by Analytic Technique and Program Dosage

	CPC groups					
Education Outcomes by Age 35	Prek to 3rd grade n=160	Prek to 2nd grade n=351	Prek to 1st grade n=116	Prek only or Prek +K n=277 [1]	No Prek had any follow on n=149	No participation n=345
**Dropout by age 16, %**						
Adjusted with covariates	9.6	11.8	10.8	10.3	14.9	13.7
Adjusted with attrition correction	9.9	12.0	10.7	10.4	15.0	14.1
Adjusted with selection correction	7.8	13.3	9.8	11.6	16.0	13.5
Adjusted with attrition & selection	7.9	13.3	9.8	11.6	16.1	13.7
**4-year high school graduation, %**						
Adjusted with covariates	60.1*	54.5	43.7	48.0	42.6	45.2
Adjusted with attrition correction	59.8*	53.7	43.1	47.9	41.4	44.4
Adjusted with selection correction	60.9*	52.6	43.0	45.2	42.6	44.0
Adjusted with attrition & selection	60.7*	51.6	42.2	45.0	41.0	43.1
**On time high school graduation, %**						
Adjusted with covariates	53.1*	48.1*	34.6	37.5	37.9	33.1
Adjusted with attrition correction	52.4*	47.2	34.1	37.4	36.3	32.4
Adjusted with selection correction	53.7*	45.4	34.2	35.1	38.4	31.3
Adjusted with attrition & selection	53.2*	44.4	33.5	35.0	36.8	30.6
**High school completion, %**						
Adjusted with covariates	88.8	87.5	88.4	85.5	81.4	80.0
Adjusted with attrition correction	88.4	87.2	88.5	85.7	80.3	79.7*
Adjusted with selection correction	88.2	87.4	87.1	84.8	82.2	79.3
Adjusted with attrition & selection	87.8	86.8	87.1	84.9	81.1	79.0+
**High school graduation, %**						
Adjusted with covariates	65.9*	58.3	47.2	53.2	47.8	51.8
Adjusted with attrition correction	65.5*	57.5	46.8	53.2	46.0	51.1
Adjusted with selection correction	66.3*	56.2	46.6	49.9	48.1	51.2
Adjusted with attrition & selection	65.9*	55.2	46.0	49.8	46.3	50.8
**Year of education (range 7-22)**						
Adjusted with covariates	12.97	12.96	12.59	12.65	12.22*	12.38
Adjusted with attrition correction	12.95*	12.93	12.56	12.65	12.18*	12.26
Adjusted with selection correction	12.91*	12.98	12.60	12.59	12.17*	12.35
Adjusted with attrition & selection	12.89*	12.95	12.58	12.59	12.14*	12.32
**College attendance, %**						
Adjusted with covariates	64.6	62.8	58.4	59.7	55.9	52.5
Adjusted with attrition correction	63.9	62.2	58.7	59.6	55.4	52.1
Adjusted with selection correction	63.3	62.5	57.6	58.3	54.3	51.4
Adjusted with attrition & selection	62.5	61.7	57.7	58.1	53.6	50.7
**4-year college attendance, %**						
Adjusted with covariates	30.5	31.3	22.4	29.2	20.9	22.4
Adjusted with attrition correction	30.3	30.6	22.4	29.1	20.1*	22.0
Adjusted with selection correction	27.9	31.3	22.6	28.2	20.7	22.8
Adjusted with attrition & selection	27.5	30.6	22.3	28.3	20.0*	22.4
**Associates' degree or higher, %**						
Adjusted with covariates	18.3	18.4	11.7	15.2	9.7	11.9
Adjusted with attrition correction	17.7	17.9	11.9	14.8	9.4*	11.3
Adjusted with selection correction	16.2	18.6	12.3	13.9	7.0*	10.7
Adjusted with attrition & selection	15.5	18.1	11.9	13.6	6.8*	9.9
**Bachelors' degree or higher, %**						
Adjusted with covariates	12.3	15.1*	9.2	8.7	6.1	8.3

	CPC groups					
Education Outcomes by Age 35	Prek to 3rd grade n=160	Prek to 2nd grade n=351	Prek to 1st grade n=116	Prek only or Prek +K n=277 [1]	No Prek had any follow on n=149	No participation n=345
Adjusted with attrition correction	12.1	14.8	8.8	8.8	6.0	8.0
Adjusted with selection correction	11.3	15.9*	9.8	8.6	4.8	7.7
Adjusted with attrition & selection	11.0	15.4*	9.5	8.5	4.7	7.4
**Masters' degree or higher, %**						
Adjusted with covariates	--	--	--	--	--	--
Adjusted with attrition correction	--	--	--	--	--	--
Adjusted with selection correction	--	--	--	--	--	--
Adjusted with attrition & selection	--	--	--	--	--	--
**Postsecondary Credential, %**						
Adjusted with covariates	26.6	23.5	16.2	19.8	15.1	18.8
Adjusted with attrition correction	26.0	23.2	16.1	19.3	14.6	18.5
Adjusted with selection correction	27.7*	24.3	16.4	18.0	12.5	18.0
Adjusted with attrition & selection	27.3*	24.0	15.8	17.7	12.2	17.9

Note. Gender, race, and sociodemographic factors are included as covariates. Child welfare history by age 4 is not included in the models of Bachelors' degree because it predicted the outcomes perfectly. There are 57 cases (4%) report Masters' degree or higher. The adjusted means by the 6 groups are too small to report. When examined by the 4 groups, the "no prek and any school-age group" predicted the outcome perfectly, so that group was not included in the analysis. 1. Reference group for all comparisons.
*95% CI does not include zero.

eMethods. Study Background, Measures, and Analysis

1. Study Background

The Chicago Longitudinal Study (CLS) is a prospective cohort investigation of early childhood experiences and well-being over the life course (1-3). The study sample of 1,539 children of the same age attended early childhood programs in the Chicago Public Schools over 1983-1986, and have been followed to midlife. Nearly two thirds (N = 989) attended the Child-Parent Center (CPC) Education Program in preschool and kindergarten and one third (N = 550) attended alternative kindergarten programs in the Chicago Public Schools. All children completed kindergarten in the spring of 1986, with those continuing in CPC up to 2nd or 3rd grade. CLS participants were born in 1979 and 1980 and grew up in high-poverty neighborhoods in Chicago. Matching the racial and ethnic composition of the neighborhoods, 92.9% of the cohort is African American and 7.1% are Hispanic. Nearly 80% of participants resided in areas of concentrated poverty, defined as 60% or more of individuals at/below 185% of the federal poverty line. The high degree of economic disadvantage faced by study participants is fully described elsewhere (4, 5).

In this article, data are included from birth to age 35, up to 32 years after program enrollment at ages 3-4 years. Data have been collected from many sources and provide a full accounting of not only of educational attainment but predictors and antecedents. These include birth records, K-12 school records, parent and teacher surveys, participant surveys, involvement in the criminal justice system, employment and earnings, and school enrollment and graduation from high school through postsecondary education. The National Student Clearinghouse provides the most complete records of college degree completion, and these records are supplemented with participant reports, and data directly from institutions.

The CLS is currently in the early midlife phase and investigators are examining the links between early childhood experiences and well-being up to age 35. Previous phases of the study have investigated the individual, family, intervention and school predictors of well-being up to age 10, 15 to 18, 18 to 24, and age 26 to 28 (6-8). Samples sizes have ranged from 1,233 to 1,473, which reflect the combination of survey response and administrative records. The sample size in this article is 1,398, and includes individuals with valid data on educational attainment up to age 35. The main sources are the National Student Clearinghouse, Illinois Shared Enrollment and Graduation Consortium (which was later incorporated into the Clearinghouse), school administrative records, and self-reports.

2. Design and Validity of Program Contrasts

The CPC program group is a complete cohort of 989 children who attended preschool and kindergarten in all 20 CPCs. The comparison group of 550 children attended full-day kindergarten programs in five randomly selected schools participating in the Chicago Effective Schools Project (CESP; N = 374) or in CPC-affiliated schools (N = 176). Both interventions were for children at risk of school underachievement due to poverty and related factors. 15% of the comparison group attended Head Start preschool (3, 8). Consequently, the CLS is a matched group, alternative-intervention quasi-experimental design in which the performance of CPC participants is compared to children in demographically-similar neighborhoods who enrolled in the usual early childhood interventions available to vulnerable families in the Chicago Public School District. School-age services from first to third grades were available to all children who enrolled in the CPC-affiliated schools regardless of preschool or kindergarten participation. eTable 1 shows the characteristics of the original sample and by attrition status by age 35.

The comparison group matched the program group on age, eligibility and participation in intervention, and neighborhood and family poverty. Eligibility criteria for CPC and CESP enrollment were similar and included (a) residence in a school area receiving federal education aid from Title I of the Elementary and Secondary Education Act of 1965, (b) demonstration of high educational need, and (c) parents agree to participate in the program to support children's learning. For CPC, this was specified as being involved up to one-half day per week in the program.

Because the CPC program was a larger-scale established intervention operating in the school district since 1967, had positive evidence of effectiveness on student achievement and school success, and was reserved for those most in need, random assignment to groups was impossible. Not only would it have been unethical, as program benefits were known and not uncertain, but contamination (noncompliance) would have most surely occurred. Children and families randomly assigned to the comparison condition in this school-wide intervention could not be required to take the assignment, especially if they lived in a CPC attendance area. Nor would they have given the known benefits and the legal requirement that the program serve those most in educational need. Cross-contamination has occurred for other multi-level preschool to third grade interventions. The accumulated evidence in CLS demonstrates that findings are interpretable as

program effects, as estimates are consistent across many different model specifications, comparison group contrasts, and analytic techniques. These techniques include latent-variable structural modeling, propensity-score weighting, matching, and stratification, and alternative covariate specifications. Causal mechanisms of change have also been shown to be consistent with the theory of intervention. These findings and interpretations are fully documented elsewhere (9-12).

3. Adult Follow-Up and Group Equivalence

At an average age of 35.1 years, 90.5% (N = 1,398) of the original CLS cohort had data for educational attainment by May 31, 2015. Sample recovery rates for the program and comparison groups were, respectively, 91.4% and 89.8%. Approximately 60% of the sample resides in Illinois (based on the age 35 survey), with many others (15%) remaining in the Midwest. The study sample represents the original sample well on nearly all attributions and no evidence of selective attrition has been found in prior studies. Data that have been missing for education, health, and behavioral outcomes are accounted for measured covariates and predictors. Thus, the assumption of missing at random has been commonly found.

eTables 1 and 2 show the characteristics of age 35 follow-up and attrition sample as well as the program and comparison groups at the beginning of the project. Child and family characteristics were measured from birth records, school and health administrative records, and family surveys. The latter assessed baseline characteristics when children were primarily between 7 and 12 years; and they supplemented records data. The p-values show the significance of group differences at the beginning of the study and at follow up. For early home environment (e.g., adverse child experiences), retrospective reports from participants were used. As shown in eTable 1, the characteristics of the original and age 35 study samples are generally similar, including for the number of family risks (4.52 vs. 4.49, respectively), CPC preschool (64.3% vs. 64.7%, respectively) and extended program participation (35.9% vs. 36.8%, respectively). However, the attrition sample had a higher proportion of males compared to the follow-up sample (71.4% vs. 47.6%) and was more likely to be disadvantaged. Since the attrition sample represented only 9.2% of the original cohort, the impact of these differences was small.

As shown in eTable 2, the two consistent differences that have been found are that program participants are from higher poverty neighborhoods and mothers have higher rates of high school completion as reported in birth records. These differences are reduced once other socio-demographic factors are taken into account. However, the advantage in parent education is specific to program females, as rates of high school graduation are nearly identical for program and comparison males. Indeed, no differences between program and comparison males have been found for any baseline measure or covariate—which number over 30 indicators (eTable 2).

4. Child-Parent Center (CPC) Education Program

The CPC program is fully described in many reports (4, 5, 13). It began in 1967 in four new centers on the city's westside. This was the result of the landmark Elementary and Secondary Education Act of 1965 for which federal funding from Title I of the Act was used by the school district to open the centers. The Chicago Public School District was the first to use Title I for preschool and thereby established CPC as the second oldest (after Head Start) federally-funded preschool. Although CPC began as a comprehensive preschool program, children received continuing services in kindergarten and the early grades the following year, resulting in the preschool to 3rd grade program that it is today. The program was modified as a school reform model in 2012 as part of expansion in and outside of Chicago funded by the U. S. Department of Education. Six core elements are implemented; effective learning experiences, collaborative leadership, aligned curriculum, parent involvement and engagement, professional development, and continuity and stability. A system of resources is now available to plan, monitor, and evaluate progress for wide-scale use and sustainability. This includes a program manual for implementation (14).

The program was developed in response to three major problems facing Chicago schools: low rates of attendance, family disengagement with schools, and low student achievement. The conceptual foundation is that well-being is a product of proximal and distal influences at multiple levels of contexts (individual, family, school, community) experienced during the entire early childhood period (ages 3 to 9). The program's focus on the quality and continuity of learning environments indicates that optimal development can be promoted through enriched experiences and settings during early childhood and the transition to school. Due to discontinuities in instructional support and philosophy between early childhood and school age settings, improvements in the integration and alignment of services during this important ecological transition improves children's levels of readiness for kindergarten that are sustained over the elementary grades.

CPC provides comprehensive education and family support services. Under the direction of a leadership team at each site and in collaboration with the Principal, CPC enhances school readiness skills,

increases early school achievement, and promotes parent involvement. It is a stand-alone school or center in which all children receive services. Health and nutrition as well as referrals to social services also are provided. The Head Teacher (HT) or Director works under the leadership of the elementary school Principal. HTs are the administrative leads for the program and manage implementation, provide coaching and supervision to staff, and help establish expectations of performance. The Parent Resource Teacher (PRT) directs the CPC's parent resource room and family services, and outreach activities are organized by the School-Community Representative (SCR). Health services are coordinated between the preschool and elementary grades.

After preschool participation at ages 3 and/or 4 in small classes with student-teacher ratios of 17:2, the K–3rd component provides reduced class sizes (maximum of 25), teacher aides for each class, continued parent involvement opportunities, and enriched classroom environments for strengthening language and literacy, math, science, and social-emotional skills. The key program elements are described in further detail (4, 5).

5. Educational Attainment Measures by Age 35

Data were obtained from Chicago Public School (CPS) yearly up to 2003, Illinois Department of Child and Family Services (DCFS) in 2003 and 2008, Illinois Shared Enrollment and Graduation Consortium (ISEG) yearly from 2002 to 2009, City colleges of Chicago in 2001-2002, Illinois GED testing in 2006, the National Student Clearinghouse (NSC) in May 2014, and were supplemented by self-reports at ages 20, 22-24 and 34-35, and tracking. The cut-off date on educational outcomes is May 31, 2015 when participants were at average age of 35.1 years. All outcomes are measured dichotomously except years of education.

Data Sources	Period or time of received data	Available number of participants	Main source of age 35 outcomes	Percent of main source of age 35 outcomes
ISEG	2002-2009	560	290	20.7
NSC	May, 2014	352	247	17.7
Age 20 survey	2000-2001	708	113	8.1
Age 22-24 adult survey	2002-2004	1,142	158	11.3
Age 35 adult survey	2012 August-May 2015	429	115	8.2
CPS	March, 2003	1,439	108	7.7
DCFS	2003, and August, 2008	1,354	121	8.7
IL GED Testing	July 2006	180	104	7.4
City Colleges of Chicago	2001-2002	511	6	.4
Tracking	2000-2015	N/A	40	2.9
Two adult surveys			7	.5
ISEG and one adult Survey			21	1.5
ISEG and tracking			7	.5
NSC and age 34 adult survey			61	4.4
Total			1,398	100.0

For high school completion status, 114 participants reported postsecondary education attendance, but they are missing on whether they completed high school via diploma or GED. Their types (high school graduation or GED) of high school completion were estimated based on available information from other data sources, including ISEG, NSC, CPS, and DCFS. For years of education, 2 participants are missing last grade they completed before they dropped out of school.

Across all sources of information, 72% of the age 35 follow-up sample had available administrative records from one or more sources, with the remainder from survey reports. These percentages were similar by intervention status: preschool (73%), comparison (70%), extended intervention to 2nd or 3rd grade (74%), and less extended participation (0 to 3 years; 70%).

The sample size for educational outcomes at age 35 is 1,398. Among the 1,539 participants in the original sample, we have educational information on 1,473 individuals. Participants fit the following criteria to be included in the age 35 sample of educational outcomes:

a) Included in age 29 educational sample (n=1,382)
b) Had any info from ISEG from 2002-2009 (n=560)
c) Had any info on NSC in May 2014 (n=352)
d) Completed age 22/24 survey between 2002 and 2004 (n=1,142)
e) Completed age 35 survey by 5/30/2015 (n=429)
f) Not deceased by 2002 January

Measures:

1. Dropout of school by age 16. If participants dropped out of school by age 16, they are coded 1. Otherwise, they are coded 0.
2. On-time high school graduation. Participants were supposed to graduate from high school in 1998 if they were not retained. If participants graduated from high school by August 1998, they are coded 1. Otherwise, they are coded 0.
3. High school completion. If participants completed high school either via diploma or GED, they are coded 1. Otherwise, they are coded 0.
4. High school graduation. If participants graduated from high school (via diploma), they are coded 1. Otherwise, they are coded 0.
5. 4-year high school graduation. If participants graduated from high school within 4 years after they started high school, they are coded 1. Otherwise, they are coded 0.
6. Attended college (2- or 4-year college). If participants have ever attended 2- or 4-year college, they are coded 1. Otherwise, they are coded 0.
7. Attended a 4-year college. If participants have ever attended 4-year college, they are coded 1. Otherwise, they are coded 0.
8. Certificate. Certificates/diplomas indicate the completion of a specialized program with specifically focused courses. Many occupations require a certificate, such as nursing assistant (CNAs), computer technician/software specialist, business administration and management, and carpentry. If participants completed certificates/diplomas, they are coded 1. Otherwise, they are coded 0.
9. Associates degree. If participants completed associate degrees, they are coded 1. Otherwise, they are coded 0.
10. Associate degree or higher. If participants completed associate degree or higher (bachelor, master, and doctor degrees), they are coded 1. Otherwise, they are coded 0.
11. Bachelor degree or higher, If participants completed bachelor degree or higher (master or doctor degrees), they are coded 1. Otherwise, they are coded 0.
12. Master degree or higher. If participants completed master degree or higher (doctor degree), they are coded 1. Otherwise, they are coded 0.
13. Any postsecondary credential. If participants completed any postsecondary credential, including certificate, diploma, associate degree, bachelor degree, master degree, MBA, or Doctoral degree, they are coded 1. Otherwise, they are coded 0.
14. Years of education was coded as a continuous variable, ranging from 7 to 22 (doctoral degree was coded as 22). Obtaining a GED was coded 12, and college attendance was coded depending on credits earned. Thirty credits were treated as a year of college attendance. For example, 30 earned credits was coded 13, and 60 earned credits was coded 14. The sample size was 1,396 due to missing values. Two participants dropped out of school, and there are no information on the last grade they completed.

6. Analytic Strategy

Following the approaches established in previous reports (8, 15-18), program effects were estimated by probit and linear regression in STATA statistical software (19). Only years of education was a linear outcome. For each attainment measure, the impact of CPC preschool for 1 or 2 years and CPC school-age services for 1 to 3 years were estimated simultaneously with two dichotomous variables. Participation in either component of the program was independent. The effect of CPC extended intervention from preschool to 2nd or 3rd grade (PK-3) was assessed separately with three different contrasts. The first was participation for 4 to 6 years from preschool to 2nd or 3rd grade versus all others having less extensive participation that did not include preschool (0 to 4 years; Extended group-1). The second was PK-3 versus participants with no CPC participation (0 years; Extended group-2). The third contrast was PK-3 versus participants who participated in only preschool and kindergarten (2-3 years; Extended group-3). This contrast assessed the added value of PK-3 above and beyond earlier intervention whereas Extended group-2 assesses the total impact of PK-3.

Findings are reported as adjusted rates (percentages) or means between groups after accounting for potential biases in estimation. The covariates included 15 child and family attributes. With the exception of public aid, subsidized lunches, and home environment, the covariates were measured primarily from birth to age 5 from administrative records (e.g., birth records, public aid, child welfare, neighborhood poverty) or family surveys. A dummy code for missing data on the covariates also was included in the models to assess if estimates based on multiple imputation were similar for those with and without missing data. Preprogram measures and covariates from administrative records came from the Illinois Department of Child and Family Services, the Illinois Department of Public Health, the Illinois Department of Health and Family Services, and the Chicago Public Schools. School and neighborhood poverty status were from U. S. Census records. Four models were estimated for each outcome. The unadjusted model is also included as point of reference.

Model 1 (covariate-adjusted). This is the standard probit-regression model that includes 15 covariates, including earlier (preschool) and later (school-age) intervention entered simultaneously. PK-3 intervention impacts were estimated separately. Prior studies demonstrate the consistency of this model with latent-variable, propensity score weighting, and propensity score matching (5-9). The model provides a comparison with the others

Model 2. (attrition correction). This specification is Model 1 estimated as a weighted regression, whereby the weight is the Inverse Probability Weighting (IPW) for attrition. In this approach (20, 21), the predicted probability of being in the recovery sample (R = 1; otherwise 0) is estimated for each measure of educational attainment via probit regression conditioned on the predictors (X) hypothesized to influence sample recovery. The propensity score was estimated as:

(1) $P_{1i} = Pr(R = 1|X) + e$

The model had 26 variables, including program participation and the covariates as predictors (see eTable 4). The inverse of this predicted probability ($1 / P_{1i}$) was used as a weight variable for the recovery sample ($1 / (1 - P_{1i})$ for the attrition sample) in all outcome models after verifying that data were missing at random (controlling for Xs) and that propensity distributions between groups overlapped. The IPW approach generally produces estimates with the lowest variances and standard errors in large samples (22, 23). Unlike propensity score matching, IPW uses all available data and can be combined with other propensity scores to estimate more complex models (23). eTables 5 to 12 and eFigures 1 to 4 show further results and supporting evidence.

Model 3. (program selection correction). Findings were also reported for models using IPW weights (P_{2i}) for selection into each of the intervention components. The program selection scores were estimated as follows, in which T denotes participation in the program (T = 1; otherwise 0) and X a set of covariates:

(2) $P_{2i} = Pr(T = 1|X) + e$

The estimated model for program selection (preschool, school-age, and extended intervention) was based on 17 preprogram predictors with weights $1/ P_{2i}$ for the program group and $1/(1 - P_{2i})$ for the comparison group (see eTable 3). Estimates based on propensity score matching and regression-based propensity score adjustments yielded similar results.

Model 4. (attrition and program selection correction). As the main model of the study, this specification is the IPW weighted regression with the multiplicative weight term (Equation 3: $P_{3i} = P_{2i} * P_{1i} + e$) as the weight variable. This model estimates the adjusted group differences accounting for attrition from the sample and selection into the program. The models were estimated separately for preschool/school-age and extended intervention (PK-3). See eTables 5 to 12. Subgroup results are shown in eTables 7 to 10 and eFigures 3 and 4.

The validity of each model in producing estimates interpretable as program (causal) effects is based on three major assumptions that have been met in this and prior studies (4-8, 15-18). First, participation and selection into the program is unaffected by the services other participants receive. This absence of treatment contamination or cross-over between program and control conditions is called the stable-unit-treatment-value assumption. Second, participation or selection in the program (T) is conditionally independent (given covariate Xs) of the response or outcome (Y) of intervention (i.e., $Pr(T/X, Y) = Pr(T/X)$. That is, after

accounting for the observed (measured) predictors of participation, the estimated propensity score for each individual is uncorrelated with one or more outcomes. Thus there is no specification error or omitted variables that are jointly correlated with program participation and outcome. A corollary assumption is that the distribution of propensity scores for each program group shows common support in that they are predominately overlapping (e.g., have similar propensities for participation or attrition). Supporting evidence for these three assumptions indicate that strongly ignorable treatment assignment was present, which is a functionally similar to groups being randomly assigned to the program (24, 25).

To enhance interpretability, coefficients from probit regression were transformed to marginal effects. 95% confidence intervals were reported. Subgroup effects were highlighted for outcomes showing overall main effects. See eTables 5 to 12 and eFigures 1 to 4 for results. Clustering by 25 program and control sites at the beginning of the intervention made no difference in estimates (intra-class corrections averaged .05 based on a two-level unadjusted variance components model). This is not surprising given the length of time between participation and outcome measurement.

References

1. Reynolds AJ. Educational success in high-risk settings: Contributions of the Chicago Longitudinal Study. *J School Psychol*. 1999, *37*, 345.
2. Chicago Longitudinal Study. *Chicago Longitudinal Study: User's guide*. (University of Minnesota, Institute of Child Development, Minneapolis, 2005).
3. Reynolds AJ, Ou S. Early childhood to young adulthood: An introduction to the special issue. *Child Youth Serv Rev*. 2010; *32*: 1045.
4. Reynolds AJ. *Success in early intervention : the Chicago child parent centers*. Child, youth, and family services. (University of Nebraska Press, Lincoln, Neb., 2000)..
5. Reynolds AJ, Temple JA, White BA, Ou S, Roberston DL. Age 26 cost-benefit analysis of the Child-Parent Center early education program. *Child Dev*. 2011; 82: 379.
6. Ou S, Reynolds AJ. Early childhood intervention and educational attainment: Age 22 findings from the Chicago Longitudinal Study. *Journal of Education for Students Placed at Risk (JESPAR)*. 2006; *11*: 175-198.
7. Reynolds AJ, Temple JA. Extended early childhood intervention and school achievement: Age thirteen findings from the Chicago Longitudinal Study. *Child Dev*. 1998; *69*, 231.
8. Reynolds AJ, Temple JA, Ou S, Arteaga IA, White BA. School-based early childhood education and age-28 well-being: Effects by timing, dosage, and subgroups. *Science*. 2011; *333*: 360-364.
9. Reynolds AJ, Temple JA. Quasi-experimental estimates of the effects of a preschool Intervention: Psychometric and econometric comparisons. *Evaluation Rev*. 1995*;* 19: 347-373.
10. Reynolds AJ. Confirmatory program evaluation: Applications to early childhood interventions. *Teachers College Record*. 2005; *107:* 2401.
11. Reynolds AJ, Ou S, Topitzes JW. Paths of effects of early childhood intervention on educational attainment and delinquency: a confirmatory analysis of the Chicago Child-Parent Centers. *Child Dev*. 2004; *75*: 1299.
12. Reynolds AJ, Ou S. Paths of effects from preschool to adult well-being: A confirmatory analysis of the Child-Parent Center Program. *Child Dev*. 2011; 82, 555-582.
13. Sullivan LM. *Let us not underestimate the children*. (Scott Foreman & Co., Glenview, IL, 1971).
14. Reynolds AJ, Hayakawa M, Candee AJ, Englund MM. *CPC P-3 Program Manual: Child-Parent Center Preschool-3rd Grade Program*. (University of Minnesota, Minneapolis, 2016).
15. Reynolds AJ, Temple JA, Roberston DL, Mann EA. Age 21 cost-benefit analysis of the Title I Chicago Child-Parent Centers. *Ed. Eval. & Policy Analysis*. 2002; *24:* 267-303.
16. Temple JA, Reynolds AJ. Benefits and costs of investments in preschool education: Evidence from the Child-Parent Centers and related programs. *Economics of Education Review*. 2007; *26*, 126.
17. Reynolds AJ, Temple JA, et al. Effects of a school-based, early childhood intervention on adult health and well-being: A 19-year follow-up of low-income families. *Arch Pediat Adol Med*. 2007; *161:* 730-739.
18. Reynolds AJ, Temple JA, Robertson DL, Mann EA. Long-term effects of an early childhood intervention on educational achievement and juvenile arrest: A 15-year follow-up of low-income children in public schools. *JAMA*. 2001; *285*: 2339-2346.
19. StataCorp, *Stata: Release 14*. (StataCorp LP, College Station, TX, 2015).

20. Cummings P. Propensity scores. *Arch Pediatr Adolesc Med*. 2008; *162*, 734.
21. Imbens GW, Wooldridge JM. Recent developments in the econometrics of program evaluation. *Journal of Economic Literature*. 2009; *47*, 5.
22. Hirano K, Imbens GW, Ridder G. Efficient estimation of average treatment effects using the estimated propensity score. *Econometrica*. 2003; *71,* 1161.
23. Kurth T, et al. Results of multivariable logistic regression, propensity matching, propensity adjustment, and propensity-based weighting under conditions of nonuniform effect. *Am J Epidemiol*. 2006; 163: 262.
24. Robins JM, Hernan MA, Brumback B. Marginal structural models and causal inference in epidemiology. *Epidemiology*. *2000; 11*, 550.
25. D. B. Rubin. For objective causal inference, design trumps analysis. *Annals of Applied Statistics*. 2008; *2*(3): 808-840.

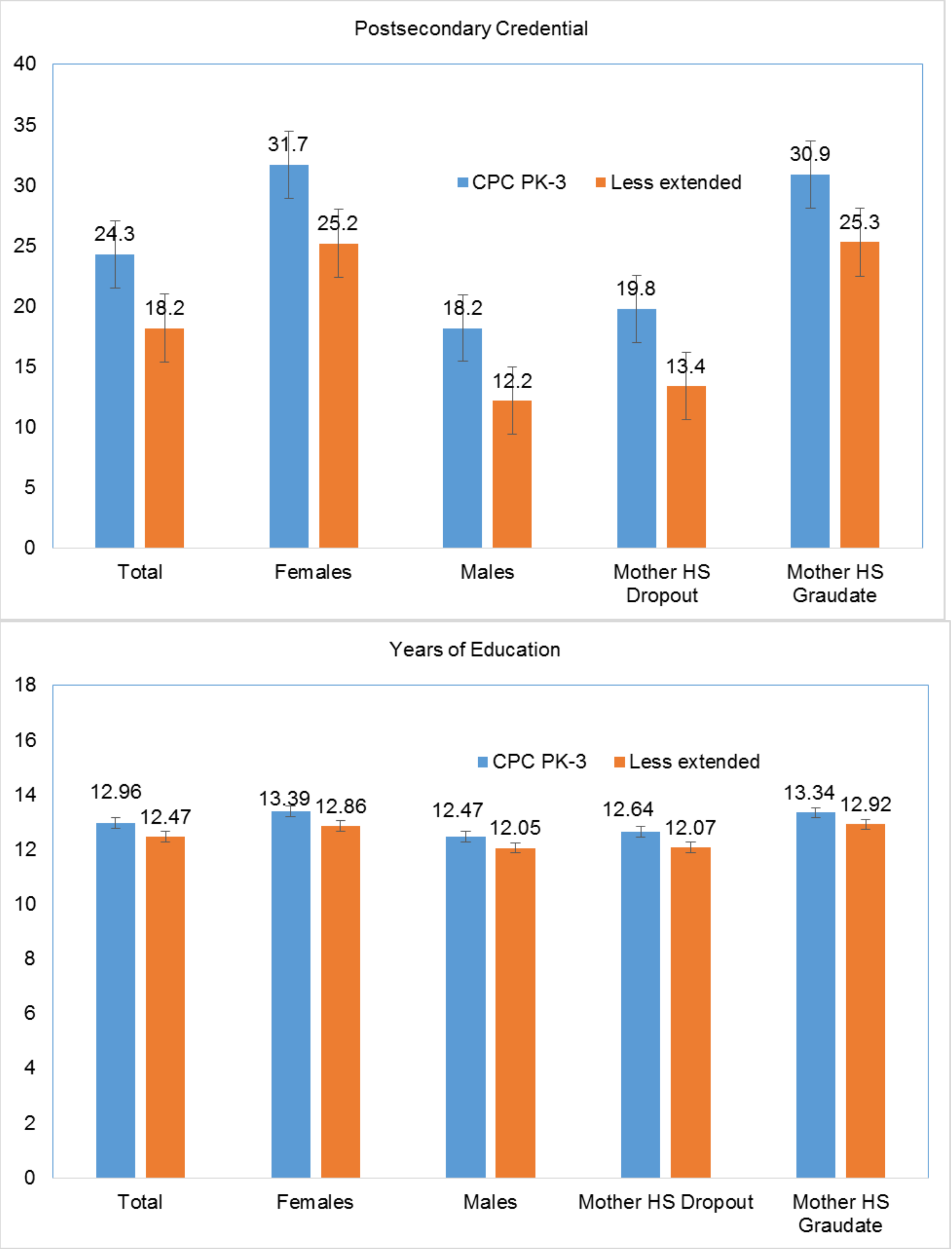

eFigure 1. Adjusted Rates for Two Measures of Attainment by Preschool to Third Grade Participation (4-6 Years)

Values are marginal rates adjusted for Inverse Probability Weighting (IPW) for program selection and attrition

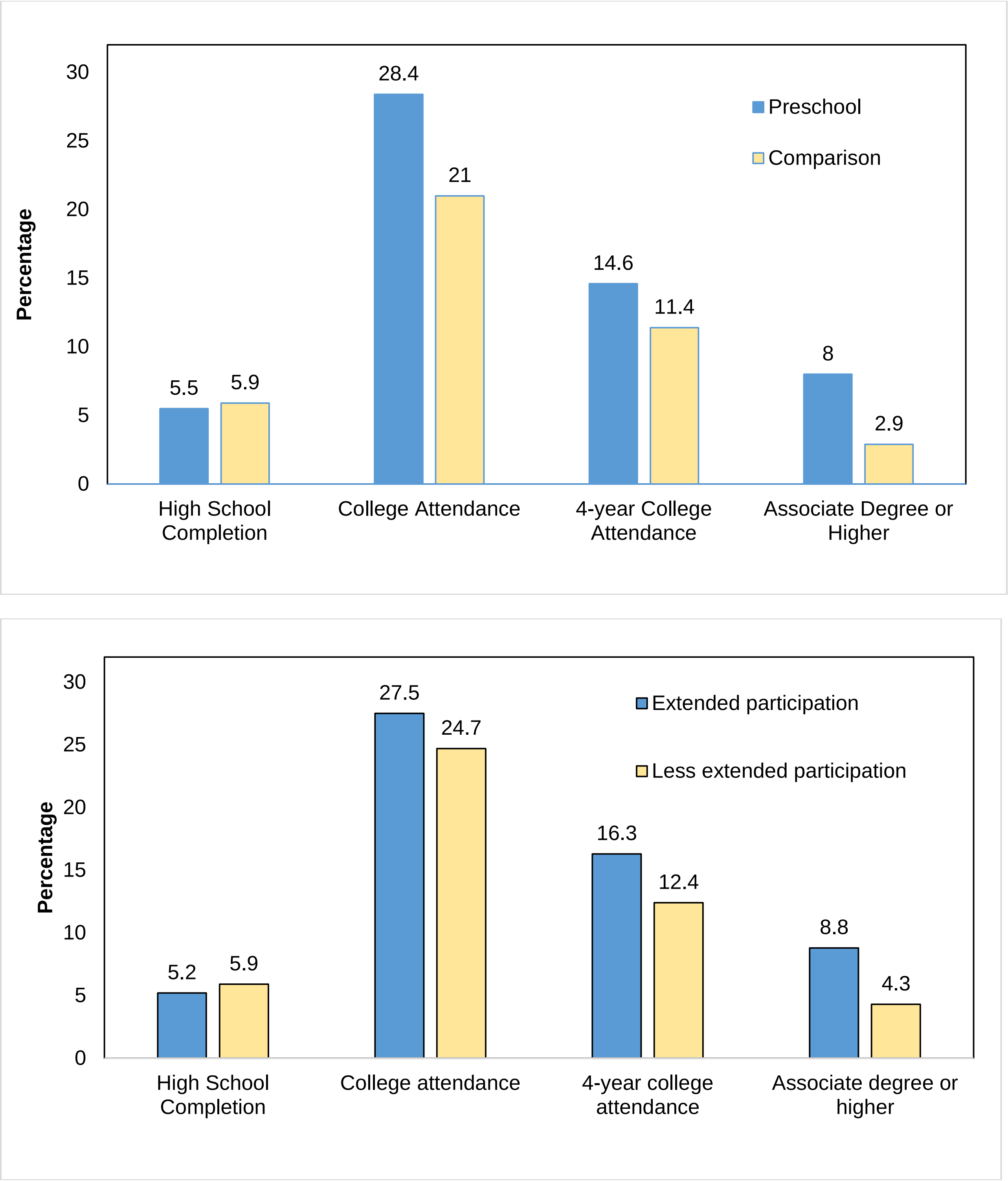

eFigure 2. Rate Difference on Outcomes Between Ages 28 and 35 by CPC Preschool Group

This figure shows the unadjusted rate difference in high school completion, college attendance, 4-year college attendance, and Associate Degree or higher, between ages 28 and 35 by CPC preschool and extended groups.

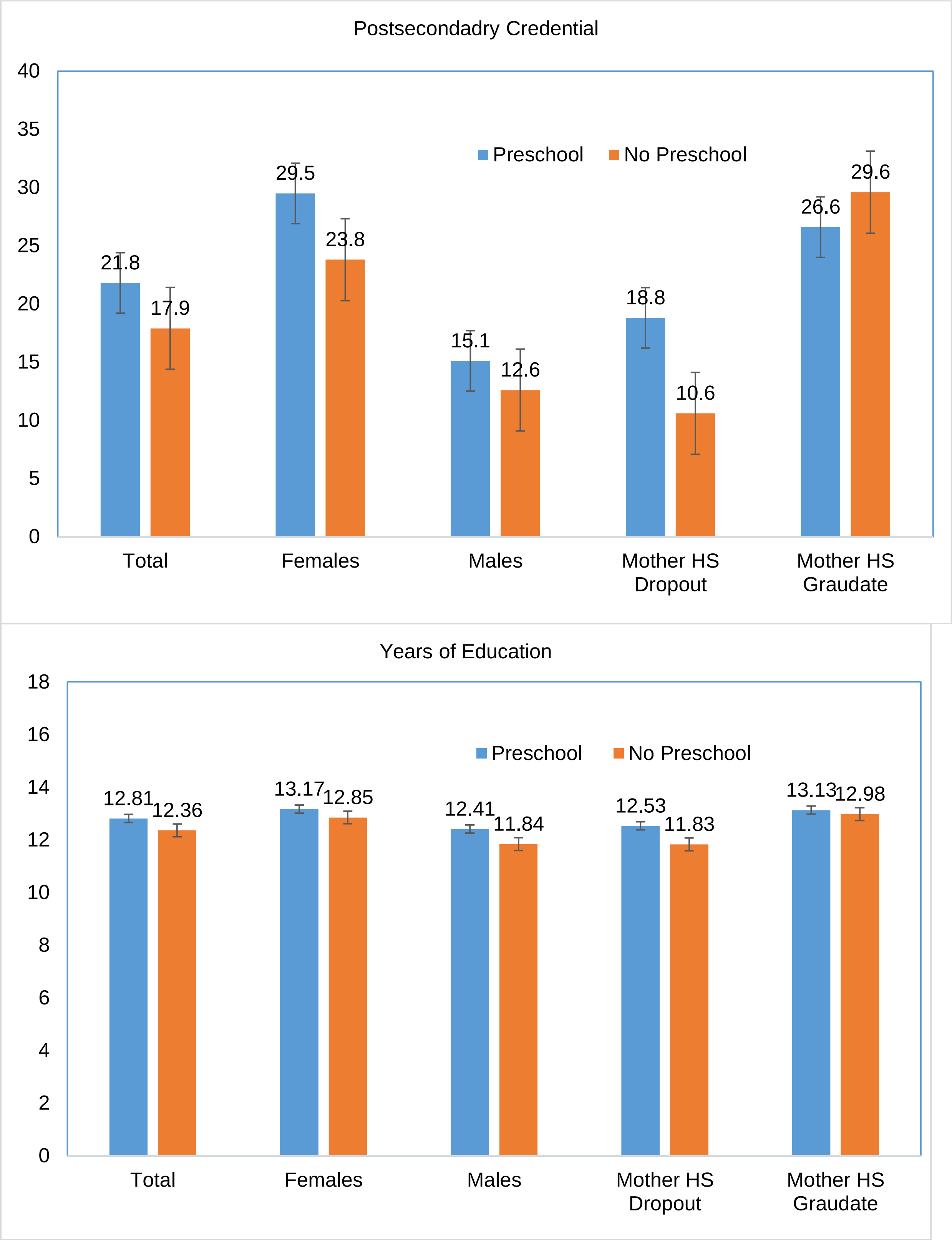

eFigure 3. Adjusted Rates for Two Measures of Attainment by CPC Preschool Participation

Values are marginal rates adjusted for Inverse Probability Weighting (IPW) program selection and attrition. CPC school-age participation was also included in the model.

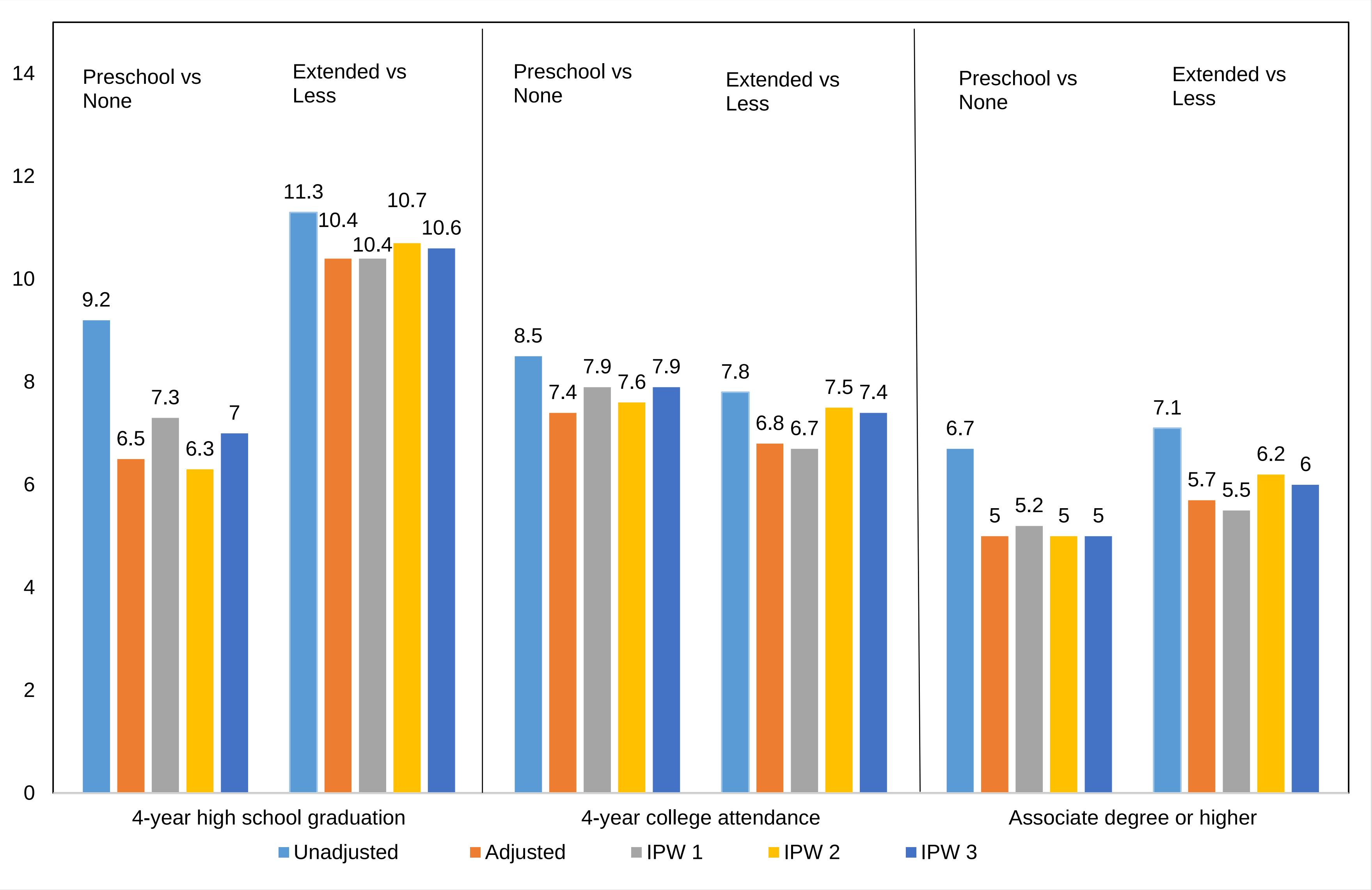

eFigure 4. Propensity-Score Weighted Differences by Program Groups

Differences between program and comparison groups for 3 outcomes and 5 analytic models: (a) unadjusted, (b) adjusted for covariates in Table 2, (c) adjusted for covariates and attrition through Inverse Probability Weighting (IPW 1), (d) adjusted for IPW selection into program by preschool and extended groups (IPW 2), and adjusted for both IPW selection into the program and IPW attrition (IPW 3). The latter is the main model for the study.
